# Multi-omic spectral clustering with the flag mean

**DOI:** 10.1093/bioadv/vbag217

**Published:** 2026-08-04

**Authors:** Charlie M Carpenter, Ziwei Tian, Jonathan K Harris, Michael Kirby, Traci Lyons, Christopher Peterson, Brandie D Wagner, Debashis Ghosh

**Affiliations:** Department of Biostatistics and Informatics, University of Colorado Anschutz Medical Campus, Aurora, CO 80045, United States; Department of Biostatistics and Informatics, University of Colorado Anschutz Medical Campus, Aurora, CO 80045, United States; Department of Pediatrics, University of Colorado Anschutz Medical Campus, Aurora, CO 80045, United States; Department of Mathematics, Colorado State University, Fort Collins, CO 80523, United States; Division of Medical Oncology, Department of Medicine, University of Colorado Anschutz Medical Campus, Aurora, CO 80045, United States; Department of Mathematics, Colorado State University, Fort Collins, CO 80523, United States; Department of Biostatistics and Informatics, University of Colorado Anschutz Medical Campus, Aurora, CO 80045, United States; Department of Biostatistics and Informatics, University of Colorado Anschutz Medical Campus, Aurora, CO 80045, United States

## Abstract

**Motivation:**

One common goal in multi-omics studies is to identify subgroups within the study’s cohort. Many methods create subgroups through unsupervised clustering, and, to our knowledge, all of these methods attempt to infer a common subspace or latent cluster across multiple views. We argue that this is a strict assumption that may not truly exist in many datasets.

**Results:**

We use the classic spectral clustering algorithm in conjunction with the flag manifold. Together, this allows for differing cluster structures across the omics profiles, leading to a novel approach for more flexible subtyping in multi-omics studies. We study a dataset on ventilator associated pneumonia in children. These data contain airway microbiome and transcriptome. Through simulation studies, we demonstrate that the flag mean of separate clustering subspaces can accurately capture the span of a joint clustering space. It is also robust to varying noise structures and number of features across omics profiles. Our proposed method also outperforms popular multi-omics clustering methods in the presence of differing group sizes. This proposed method is general enough to apply to other multi-omics studies as well as any multi-view study that uses spectral clustering.

**Availability and implementation:**

Code for these methods are written in R and are freely available through GitHub at https://github.com/Ghoshlab/MMOC or through CRAN at https://cran.r-project.org/web/packages/MMOC/index.html.

## 1 Introduction

Clustering is a fundamental task in data science. Broadly speaking, it is the task of grouping similar objects together while separating objects that are unlike each other. Clustering is often an important first step to understanding a dataset and can be a useful hypothesis generation tool (Maimon and Rokach 2006). As the high-throughput technologies used for collecting omics data become more efficient and cost effective, multi-omic studies have become an extremely active field. Using multiple representations (omics profiles) of a single subject is important to understand them fully. In theory, each omics dataset is giving complementary information about the subject. In recent years many unsupervised clustering algorithms for multi-omic data have been produced.

Multi-omic clustering algorithms often focus on generalizing spectral clustering. These approaches have been applied to multi-view studies (De Sa 2005, Kumar *et al*. 2011, Xia *et al*. 2014, Chikhi 2016) and multi-omics studies (Mitra and Saha 2019, Mitra *et al*. 2020). Random walk based similarities have also been incorporated into these generalized spectral clustering algorithms (Zhou and Burges 2007, Xia *et al*. 2014). Multiple kernel learning has also been successfully applied to this task (Speicher and Pfeifer 2015). Probabilistic model approaches cluster under the assumption that the data from each view come from a low dimensional distribution and cluster on an estimated mixture of these distributions. These methods find the maximum likelihood of an assumed model based on the data and perform k−means on this lower dimensional representation (Shen *et al*. 2009, Lock and Dunson 2013, Rohart *et al*. 2017, Nguyen *et al*. 2019, 2017, Argelaguet *et al*. 2020). Tensor based methods are likely the most active area in imaging applications. These methods usually arrange the graph Laplacian of each view into a higher order matrix (tensor) and apply tensor decomposition approaches to find low rank embeddings of the data (Xia *et al*. 2014, Zhang *et al*. 2017, Gao *et al*. 2020, Jia *et al*. 2020, Zhang *et al*. 2020, 2022). Extended spectral clustering approaches aim to extend the standard and well known spectral clustering algorithm (Ng *et al*. 2002, von Luxburg 2007) to multi-view settings. These involve applying the standard algorithm to a pooled estimate of the Laplacian or encouraging information sharing through a more robust objective function (De Sa 2005, Zhou and Burges 2007, Nepusz *et al*. 2010, Kumar *et al*. 2011, Zhang *et al*. 2015, Chen *et al*. 2017, John *et al*. 2020). Finally, manifold clustering techniques take advantage of algorithms developed for optimization over manifolds to handle the complex spaces of their objective functions (Wei *et al*. 2010, Yong Wang *et al*. 2011, Zong *et al*. 2017, Carson *et al*. 2017, Yu *et al*. 2019, Khan and Maji 2021a,b).

Historically, many early and influential multi-view clustering methods were motivated by the existence of a single, unified clustering structure shared across all views, treating view-specific variation as nuisance that should be reconciled during fusion. Canonical examples include co-regularized spectral clustering (Kumar *et al*. 2011) and graph-based fusion methods such as Similarity Network Fusion (Wang *et al*. 2014). While this “rigid consensus” perspective can be effective when views are strongly correlated, it may fail to capture heterogeneity that is biologically meaningful in multi-omics studies, where different molecular layers can reflect distinct (yet valid) stratifications of samples.

In response to this limitation, recent multi-view clustering research has increasingly emphasized frameworks that explicitly relax the requirement of identical cluster assignments across views. One class of methods learns view-specific representations (or spectral embeddings) and then extract a joint representation by optimizing an alignment or consensus criterion, rather than assuming a common group structure a priori. In the subspace-clustering literature, Consistent and Specific Multi-view Subspace Clustering (CSMSC) decomposes the learned self-representation into a shared “consistent” component and a view-specific component, thereby separating information that should be conserved across views from view-unique structure (Luo *et al*. 2018). Several new methods disentangle shared and view-specific factors via information-theoretic regularization or architectural separation in deep models, learning a joint latent space that captures cross-view agreement while preserving modality-specific variation. For instance, multi-view variational approaches such as Multi-VAE explicitly model view-common and view-peculiar latent variables to separate shared biology from view-specific effects (Xu *et al*. 2021). In oncology-oriented integration, deep autoencoder frameworks have also imposed explicit orthogonality constraints between shared and specific representations to obtain robust subtyping under heterogeneous omics signals (Chen *et al*. 2023).

Another set of methods estimates multiple, contradictory-yet-meaningful clustering structures, and therefore a single consensus clustering may be scientifically limiting. Multi-View Multiple Clustering (MVMC) formalizes this perspective by seeking multiple diverse clustering solutions and using dependence penalties (e.g. HSIC-based regularization) to encourage distinct clusterings rather than collapse them into one partition (Yao *et al*. 2019). These methods assume that that “view disagreement” can represent true biological plurality (e.g. immune microenvironment versus intrinsic tumor state) rather than measurement noise.

An alternative approach has been to treat view-specific embeddings themselves as points on a geometric space and to define consensus through distances between subspaces. Concretely, they learn view-specific spectral embeddings $U_{v}$ and then estimate a joint subspace *U* that minimizes a discrepancy between $U_{v}$ and *U*, where the discrepancy may be linear (e.g. Frobenius-norm distances between projection matrices) or nonlinear (e.g. geodesic distances on Grassmann/Stiefel-type manifolds). Multi-View Clustering via Manifold Optimization (MVCMO) is an example that formulates consensus spectral clustering directly on the Stiefel manifold and uses Riemannian optimization to respect the intrinsic geometry of embedding constraints (Yu *et al*. 2019). More recently, geometric ideas have also been adopted in scalable deep settings: anchor-based deep multi-view clustering methods learn representations guided by a reduced set of anchors to approximate global structure while avoiding quadratic costs, enabling clustering at substantially larger scale (Wang *et al*. 2025).

Deep contrastive learning has further strengthened the “view-specific then align” viewpoint by defining agreement through alignment in representation space rather than requiring identical view-wise cluster structure. As one example, Simple Contrastive Multi-view Clustering (SCM) advocates a data-level fusion strategy that uses a shared encoder over concatenated, normalized multi-view inputs, coupled with multi-view data augmentation and instance-discriminative contrastive objectives to learn discriminative representations for clustering, while reducing the complexity of maintaining separate per-view encoders (Luo *et al*. 2024). These developments collectively show that modern multi-view clustering does not universally assume a strictly common group structure across views; instead, current methods often balance consistency with view specificity, and increasingly define “consensus” at the level of representations or subspaces rather than at the level of hard cluster labels.

Despite this progress, there remains a need for principled, lightweight methods that (i) operate directly on the spectral subspaces that underpin graph-based clustering, (ii) accommodate the practical reality that different omics layers may support different numbers of clusters and different signal-to-noise regimes, and (iii) provide a mathematically transparent definition of consensus that is compatible with non-Euclidean subspace geometry. Flag manifolds have seen use in extensions to PCA and subspace analysis (Pennec 2018), but to our knowledge they have not been leveraged as an organizing geometry for multi-omic clustering.

We propose a combination of the flag manifold (Monk 1959) and the classic spectral clustering algorithm (Ng *et al*. 2002, von Luxburg 2007). To our knowledge, there has not been any work combining these two tools or any applications of the flag manifold for multi-omic clustering. Our proposed method can be used to cluster two or more omics datasets without the assumption that every dataset contains the same number of clusters. We motivate the use of our method on two datasets: (i) a multi-omics dataset from mechanically ventilated children at risk for ventilator associated pneumonia; (ii) breast cancer datasets from the Cancer Genome Atlas (TCGA). Our proposed approach allows for more flexibility in unsupervised multi-omics clustering by allowing different omics data sources to have different clustering structures. We believe this is useful in a wide range of biomedical applications and general multi-view studies.

## 2 Methods

### 2.1 Spectral clustering

Spectral clustering is a well-known method for subspace clustering (Ng *et al*. 2002, von Luxburg 2007). Given a dataset, $X\in R^{n\times p}$, where *n* is the number of subjects and *p* is the number of biological features, one calculates an n×n symmetric matrix, *A*, whose entries measure some aspect of pairwise similarity or affinity between the columns of *X*. This matrix can be identified with a weighted graph with each subject a node and each pairwise similarity/affinity weighting the edge between the nodes. Next, one calculates the *graph Laplacian*, $L\in R^{n\times n}$ associated to *A*. There are many different strategies for building *A* and there are a handful of different formulas for associated graph Laplacians; most of them will give our algorithms equivalent information. We choose to use Ng’s construction of the graph’s normalized Laplacian


$$L=D^{-1/2}AD^{-1/2},$$

where *D* is an n×n diagonal matrix with the row sums of *A* along the diagonal. The graph Laplacian, *L*, is a positive semidefinite matrix thus the eigenvalues are all real and non-negative. These can be ordered from smallest to largest or vice versa leading to a corresponding ordering of the associated eigenvectors. The first *k* normalized eigenvectors, $u_{1},u_{2},\ldots ,u_{k}$, of *L* are collected and combined to form the matrix $U=[u_{1},u_{2},\ldots ,u_{k}]\in R^{n\times k}.$ This can then be used to find if distinct groups exist within the data using techniques, such as *K*-means, applied to the *rows* of *U*. While it is common to row normalize *U* before estimating the final clusters, this may introduce unwanted scaling into each view’s clustering space that would impact the final joint clustering, so we do not row normalize.

### 2.2 Embedded submanifolds

We will now define a class of special manifolds that have found applications to many problems involving subspaces of a fixed vector space. We are specifically interested in the class of Riemannian manifolds known as *flag manifolds* (Monk 1959). As a warm up, we give a brief description of the subclass of flag manifolds known as *Grassmann* manifolds as they help to conceptualize the flag manifolds.

The real Grassmann manifold, Gr(k,n), is a manifold whose points parameterize *k*-dimensional subspaces of the vector space $R^{n}$. Consider the space $R^{n\times k}$, which consists of all n×k matrices with real entries, and let the open submanifold of all full rank n×k matrices be denoted ${\left(R^{n\times k}\right)}^{o}$ For each $U\in {\left(R^{n\times k}\right)}^{o}$, the column space of *U*, denoted *[U]*, is then a point on Gr(k,n). In other words, given the vector space $R^{n}$, a point on the Grassmann manifold Gr(k,n) represents the span of an n×k matrix *U* rather than *U* itself. Due to this observation, two different n×k matrices having the same span represent the same point on Gr(k,n).

The real flag manifold $\mathrm{Flag}(n_{1},\ldots ,n_{p};n)$ is a manifold whose points parameterize nested sequences of subspaces of $R^{n}$ of the form


$$\{0\}⊊V_{1}⊊V_{2}⊊\ldots ⊊V_{p}⊊R^{n}$$

with the dimension of $V_{i}$ equal to $n_{i}$ for each *i*. The data $\left(n_{1},\ldots ,n_{p};n\right)$ is called the *signature* of the flag manifold. Given any increasing sequence of *p* natural numbers, $0<n_{1}<n_{2}<\ldots <n_{p}<n$, $\mathrm{Flag}(n_{1},\ldots ,n_{p};n)$ is a smooth Riemannian manifold. Figure 1 illustrates a point on Flag(1,2,3,…,k;n). Flag manifolds whose signature is a complete list of consecutive integers from 1 to *n* are called *complete* flag manifolds. Note that many applications do not require complete flag manifolds, i.e. we may allow non-consecutive integers in the signature. Flag manifolds may be understood as a generalization of Grassmann manifolds (whose points are flags are of the form $0⊊V_{1}⊊R^{n}$). Since a point on a flag manifold represents a collection of nested subspaces, we can think of the point as lying on a product of Grassmannians. For instance, a point $\mu \in \mathrm{Flag}(n_{1},n_{2},n_{3};n)$ corresponds to a point on $Gr(n_{1},n)\times Gr(n_{2},n)\times Gr(n_{3},n)$. The nesting constraint leads to $\mathrm{Flag}(n_{1},n_{2},n_{3};n)$ being realized as a proper submanifold of $Gr(n_{1},n)\times Gr(n_{2},n)\times Gr(n_{3},n)$.

**Figure 1:**
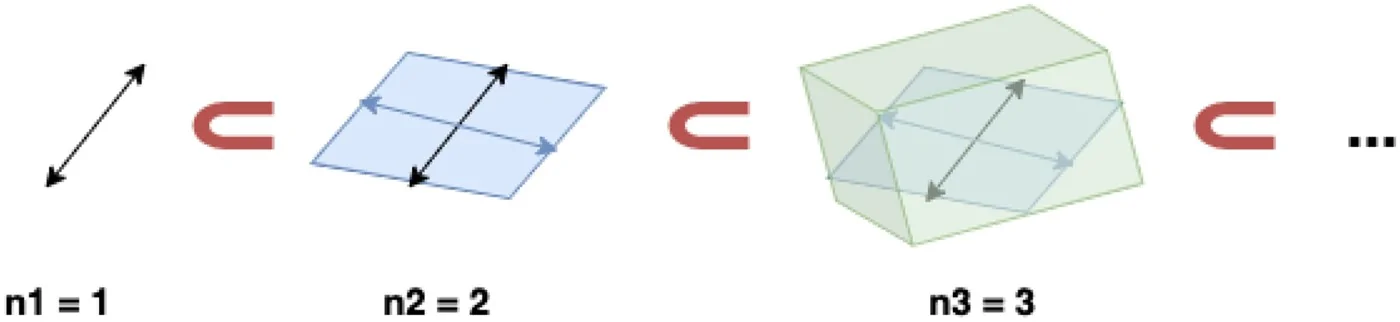
A visualization of a point in Flag(1,2,3,…,k;n).

Given a full rank n×k matrix *U*, the $i^{th}$  *sequential column space* of *U* is the span of the first *i* columns of *U*. Using Gram-Schmidt orthogonalization, it is easy to produce from *U* another full rank n×k matrix $U^{\prime }$ whose sequential column spaces are the same as the sequential column spaces of *U* but whose columns are orthonormal. Each point on Gr(k,n) or $\mathrm{Flag}(n_{1},n_{2},\ldots ,n_{k};n)$ can be represented by such a matrix. For example, consider the manifold Flag(2,3,5;n). We can partition the columns $u_{1},u_{2},u_{3},u_{4},u_{5}$ of a full rank n×5 matrix *U*, with orthonormal columns, as


$$\mu =\left[\left\{u_{1},u_{2}\},\{u_{3}\},\{u_{4},u_{5}\right\}\right],\;u_{i}\in R^{n}.$$

We then get the point, $\mathrm{span}\{u_{1},u_{2}\}\subset \mathrm{span}\{u_{1},u_{2},u_{3}\}\subset \mathrm{span}\{u_{1},u_{2},u_{3},u_{4},u_{5}\}\subset R^{n}$, in Flag(2,3,5;n). The first two columns span the 2D subspace. The 3rd column is orthogonal to the first two, and together they span a 3D subspace. The final two columns are orthogonal to the previous three, and together these span a 5D subspace of $R^{n}$. Note that the embedding approach we use does not require that the initial set of subspaces be nested. In particular, the operation of concatenating the orthonormal bases across subspaces leads to the nested structure. The importance of the flag manifold is in the additional information that can be associated to the subspaces in the nesting. In other words, we not only have a 5D subspace but also specific nested 2 and 3 dimensional subspaces. See Ye *et al*. (2021) for an explanation of a collection of numerical techniques that can be carried out on a flag manifold and for relationships to the Stiefel manifold.

### 2.3 The flag mean of subspaces

Assume we have a set of subspaces represented by orthogonal matrices ${\left\{U_{v}\right\}}_{v=1}^{V}$. Let $k_{v}=\dim([U_{v}])$. This set of subspaces can be considered as observations on a disjoint union of Grassmannians: ${\left\{[U_{v}]\right\}}_{v=1}^{V}\in \overset{V}{\underset{v=1}{\cup }}Gr(k_{v},n)$. The flag mean by definition is a single representative point on a flag manifold by solving, inductively, for a sequence of orthonormal vectors. Let d(μ,U) be the projected Frobenius norm, $\frac{1}{\sqrt{2}}||\mu \mu^{T}-UU^{T}|{|}_{F}$. We first find


(1) $$\begin{array}{cc} \mu_{1}=*\text{arg}{\text{min}}_{\mu |\mu^{T}\mu =1} & \sum_{v=1}^{V}d{\left(\mu ,U_{v}\right)}^{2} \end{array}$$

We solve for additional orthonormal vectors by finding


(2) $$\begin{array}{c} u_{j}=*\text{arg}{\text{min}}_{\mu |\mu^{T}\mu =1}\sum_{v=1}^{V}d{\left(\mu ,U_{v}\right)}^{2}\\ s.t.\;\mu^{T}\mu_{k}=0\;\mathrm{for}\;k<j. \end{array}$$

Fortunately, this sequential approach is only necessary to help conceptualize the flag mean. The actual algorithm does not require this sequential approach. The landmark paper from Edelman *et al*. (1998) gives an expression for d(μ,U) in terms of the principal angles between μ and *U*. More precisely, let θ(μ,U) denote the vector of principal angles between [μ] and *[U]*. In other words, $\theta (\mu ,U)=(\theta_{1}(\mu ,U),\ldots ,\theta_{k}(\mu ,U))$ with k=min{dim([μ]),dim([U])}. Let $\text{sin}(\theta (\mu ,U))=(\text{sin}(\theta_{1}(\mu ,U)),\ldots ,\;\text{sin}(\theta_{k}(\mu ,U)))$. We have $d(\mu ,U)=||\;\text{sin}(\theta (\mu ,U))|{|}_{2}$, where $||\cdot |{|}_{2}$ is the $l_{2}$ norm (Edelman *et al*. 1998). Solving for an optimal $\left[\mu^{*}]\in Gr(k,n\right)$ then becomes


$$\begin{array}{c} \mu^{*}={\text{arg}\;\text{min}}_{\mu \in R^{n\times k}|\mu^{T}\mu =I_{k}}\sum_{v=1}^{V}d{\left(\mu ,U_{v}\right)}^{2}\\ ={\text{arg}\;\text{min}}_{\mu \in R^{n\times k}|\mu^{T}\mu =I_{k}}\sum_{v=1}^{V}||\;\text{sin}(\theta (\mu ,U_{v}))|{|}_{2}^{2}\\ ={\text{arg}\;\text{max}}_{\mu \in R^{n\times k}|\mu^{T}\mu =I_{k}}\sum_{v=1}^{V}||\;\text{cos}(\theta (\mu ,U_{v}))|{|}_{2}^{2}\\ ={\text{arg}\;\text{max}}_{\mu \in R^{n\times k}|\mu^{T}\mu =I_{k}}\sum_{v=1}^{V}Tr\mathrm{ace}(\mu^{T}U_{v}U_{v}^{T}\mu )\\ ={\text{arg}\;\text{max}}_{\mu \in R^{n\times k}|\mu^{T}\mu =I_{k}}T\mathrm{race}(\mu^{T}(\sum_{v=1}^{V}U_{v}U_{v}^{T})\mu ) \end{array}$$

The solution of this expression can now be determined from the singular value decomposition (*SVD*) of $\sum_{v=1}^{V}U_{v}U_{v}^{T}$. In particular, the solution to this problem, $\mu^{*}$, is the matrix whose columns are the *k* normalized eigenvectors of $\sum_{v=1}^{V}U_{v}U_{v}^{T}$ associated to the largest *k* eigenvalues of $\sum_{v=1}^{V}U_{v}U_{v}^{T}$. Thus, we have $\mu^{*}=[\mu_{1},\mu_{2},\ldots ,\mu_{k}]$ which can then be associated to a point on a flag manifold by considering its sequential subspaces. Note that (numerical) ties may occur in the ordering of the eigenvalues leading to ambiguity in the ordering of the eigenvectors. In this setting we consider $\mu^{*}$ as a point on a non-complete flag manifold by grouping together eigenvectors that have the same (numerically) associated eigenvalues. See Draper *et al*. (2014) for a complete derivation as well as a more efficient algorithm to obtain $\mu^{*}$ as long as k<n. For instance, the paper shows that if we concatenate each $U_{v}$ to obtain $U=[U_{1}|U_{2}|\ldots |U_{V}]$, the same solution can be achieved as the first *k* left singular vectors appearing in the singular value decomposition of U. The algorithm is then as follows,

1. Collect subspaces $U_{1},\ldots ,U_{v}$ and ensure $U_{v}^{T}U_{v}=I$
2. Set $U=[U_{1}|U_{2}|\ldots |U_{V}]$
3. Find $\mu \Sigma V^{T}=\mathrm{SVD}(U)$
4. Let $\mu^{*}$ be equal to the first *k* columns of μ. Interpret the sequential subspaces of $\mu^{*}$ as a point on Flag(1,2,…,k;n).

Here $\mathrm{SVD}(U)=\mu \Sigma V^{T}$ is the singular value decomposition of the matrix $U\in R^{n\times p}$, where $p=\sum_{v=1}^{V}\text{dim}([U_{v}])$. The columns of the matrix μ are the left singular vectors of the matrix U, *V* is the transpose of the matrix whose columns are the right singular vectors of U, and $\Sigma \in R^{n\times p}$ is a “diagonal” matrix whose diagonal entries are the singular values arranged in monotone decreasing order.

### 2.4 Proposed method

We propose the use of the flag mean to estimate a joint clustering space from multiple spectral embeddings. Given *V* views, with *K* total groups, we calculate each views’ normalized Laplacian $L_{v}=D^{-1/2}AD^{-1/2}$ and the respective spectral decompositions, $L_{v}=U_{v}\Lambda_{v}U_{v}^{T}$, where the columns of $U_{v}$ are the normalized eigenvectors of $L_{v}$, and $\Lambda_{v}$ is the diagonal matrix of their corresponding eigenvalues arranged in order from largest to smallest. We use the adaptive density-aware kernel (John *et al*. 2020)


$$A_{ij}=\text{exp}\;\left(\frac{-||s_{i}-s_{j}|{|}_{2}^{2}}{\sigma^{i}\sigma^{j}(\mathrm{CNN}(s_{i},s_{j})+1)}\right)$$

to calculate our similarity matrices in each view since it has been shown to perform well on many different omics data types. Here, $s_{i}$ is the $i^{th}$ column of the data matrix and $\mathrm{CNN}(s_{i},s_{j})$ is the number of common nearest neighbors between subjects *i* and *j*. Finally, $\sigma_{i}$ is equal to $||s_{i}-s^{p}(i)|{|}_{2}^{2},$ where $s^{p}(i)$ is the $p^{th}$ nearest neighbor to subject *i* for some pre-specified *p*.

We collect the first $k_{v}$ eigenvectors of each $L_{v}$ and form the respective subspaces spanned by the columns of $U_{v}=[u_{1_{v}},u_{2_{v}},\ldots ,u_{k_{v}}]$. $L_{v}$ is a symmetric matrix, ensuring that the columns of $U_{v}$ can be chosen to be orthonormal for the flag mean. The set of *V* eigen-embeddings contains the relevant clustering information from each view. Finally, we calculate the flag mean of this set and treat the first *K* vectors of the flag mean as an estimate of the joint clustering space. We make several observations about this approach. First, the constituent components involve using graph Laplacians to represent view-specific cluster structure and to find the subspace that minimizes the distances to these points in a least-squares, or equivalently, a principal-angle sense. Finding principal angles between subspaces is a very well-studied problem in applied mathematics (Björck and Golub 1973, Knyazev and Argentati 2002), but the idea of generalizing this task across subspaces of nested and varying dimensions for multi-view learning appears new. Second, one can interpret the first component of the flag mean as capturing the common structure across views, with the remaining vectors capturing orthogonal components to the common structure. However, each component of the flag mean will in theory depend on all the views, and we have described a means of quantifying the contributions of views to flag mean components in the Section 3.3. Third, the major innovation of our proposed method is we can allow for different -omics data types to have different numbers of embeddings to allow for faithful cluster representation. As an example, suppose there are 3 views (e.g. transcript mRNA, copy number and methylation) and that we need $k_{1}=2$, $k_{2}=4$ and $k_{3}=3$ eigenvectors to capture the clustering structure in terms of transcript mRNA, copy number and methylation, respectively. Then the flag mean will treat points as existing on the Flag((2,6,9),n) manifold, whereas other methods will not easily be able to accommodate the varying dimensionality of the views (Draper *et al*. 2014).

### 2.5 Simulations

All data were generated from a multivariate Gaussian distribution (*MVN*) in R (R Core Team 2022). The underlying cluster structures, $Z\in R^{n\times p},$ were generated from MVN(μ,I), where *I* was the identity matrix and μ was a constant vector set to 0 or, for i>1, $2^{k_{i}-1}$. In other words, for data with three clusters (k=3), each third of the data would be generated from MVN(0,I), MVN(2,I), or MVN(4,I). Data with the same mean obviously come from the same cluster, so extra Gaussian noise from E∼MVN(0,τI) was added to *Z*, where τ was set to 2, 5, 7, or 10. $\tilde{Z}=Z+E$ is then treated as a single view with *k* clusters. We apply three popular multi-omics clustering methods, Spectrum (John *et al*. 2020), NEMO (Rappoport and Shamir 2019), and SNF (Wang *et al*. 2014) to each simulation setting as well. See the Supplementary Information for a detailed description of these three methods. We also explore the impacts of changing $p_{i}$ and changing τ between views within a study. See the Supplementary Information for a full description of these simulation settings. We set our sample size to 120 for each simulation and repeat each simulation setting 5000 times.

The cluster centers in $\tilde{Z}$ are all at least 2 units away from one another giving us a “minimum center distance to noise ratio” as 2/τ, which is how we group our simulation results for the first four studies. In our second study, we use real microbiome data from the ventilator associated pneumonia study instead of Gaussian noise to better approximate the noise we would see in a multi-omics study. We took the *p* microbes with the highest variance after a centered log ratio (CLR) transformation and used this as the noise matrix, *E*. These simulations were all run with *P* = 60. These initial studies were meant to assess and compare the effectiveness of our proposed methods with other popular multi-omics clustering algorithms in the presence of differing group sizes across views. We investigate 2, 3, and 4 view studies with various numbers of clusters within each view. We evaluated the clusterings based on their adjusted Rand index (ARI), F1-score, and silhouette scores.

### 2.6 Selecting *K* in flag mean

Our final simulation study investigates the ability of several methods to identify the correct number of clusters. We supplied all vectors from the flag mean to each method. The final clustering is then performed on the first *k* vectors of the flag mean, where *k* is the estimated number of clusters.

We use several of the methods built into the *NbClust* package in R (Charrad *et al*. 2012). For these methods we use the Euclidean distance within the joint space and the *k*-means method of clustering. We include the simple eigen-gap heuristic, which will identify the largest gap in the singular values or the flag mean. We also include two Ladle estimators which estimate the order of a matrix through the variance of their eigenvectors (Luo and Li 2016). Again, we investigate 2, 3, and 4 view studies. There is a variety in the settings for *k* that will be clear in the results. We set p=60 and τ=5 for all settings. This study only includes 1000 simulations, but the variability of most estimates was low enough to warrant the low number of simulations.

### 2.7 Ventilator associated pneumonia

The ventilator associated pneumonia (VAP) dataset comes from a study published in 2020 that sought to assess the relationship between lower airway microbiome and VAP. This was a multicenter prospective study of children ages 31 days to 18 years requiring mechanical ventilation for over 72 h. Daily tracheal aspirates were collected and analyzed by sequencing the 16S rRNA (microbiome) and metagenomic next-generation sequencing (transcriptomics) (Mourani *et al*. 2021, Mick *et al*. 2023). Following de-multiplexing, metagenomic sequencing reads were pseudo-aligned with kallisto42 (v. 0.46.1; including bias correction) to an index consisting of all transcripts associated with human protein coding and long non-coding RNA genes (ENSEMBL v.99). We excluded samples with less than 500 000 estimated counts associated with transcripts of protein-coding genes. Gene-level counts were generated from the transcript-level abundance estimates using the R package *tximport43*, with the scaledTPM method. Initial specimens were collected within 24 h of intubation, and subsequent samples were collected daily until the first attempted extubation or for up to 14 days. Microbiome and metagenomics data were only available from the first sample after intubation on all subjects.

All data were cleaned, normalized, and imputed in R (R Core Team 2022). Transcripts with ≥%20 missingness were removed from the analysis. Microbes without ≥%0.1 relative abundance in at least one subject were removed, and microbes that weren’t present in at least 5% of subjects were removed. We used the variance stabilizing transformation with a local regression fit from DESeq2 to normalize the transcriptomics (Love *et al*. 2014). Microbiome data were all normalized using the CLR transformation. These data have 36 816 transcripts and 176 microbes. Two outliers were found and removed in the transcriptomics data, resulting in 215 subjects.

### 2.8 The cancer genome atlas program

We additionally analyzed a breast cancer multi-omics cohort from The Cancer Genome Atlas (TCGA). We used the processed TCGA “multi-omics cancer benchmark” datasets assembled for systematic comparisons of multi-omics clustering methods (Rappoport and Shamir 2018), which include three omics layers: gene expression, DNA methylation, and miRNA expression, along with clinical annotations and survival outcomes.

All data were cleaned and normalized in R. For each omics matrix, we retained only primary tumor aliquots, converted TCGA aliquot barcodes to patient IDs, and collapsed technical replicates/multiple aliquots per patient to a single profile by taking the mean across aliquots. We then restricted to patients present in all omics views by intersecting patient ID sets across views. Within each view, we removed zero-variance features, z-scored features across patients, and retained the most variable features (top 5000 for expression, top 10 000 for methylation, and top 1000 for miRNA). Gene expression values were log-transformed with a pseudocount of one before z-scoring. For downstream evaluation, PAM50 subtype calls were taken from TCGA clinical annotations, and survival endpoints were drawn from TCGA clinical resources (overall survival [OS] and progression-free interval [PFI]); patients with missing labels or missing survival information were excluded from ARI and log-rank calculations.

## 3 Results

### 3.1 Simulations

We display simulation results for the noise parameter, τ, set to 5 and the noise coming from microbiome data in Fig. 2. Along the x-axis we describe the number of clusters simulated within each view, e.g. 2, 3, 4 represents a 3-view study with 2, 3, and 4 clusters, respectively. When data are generated with purely Gaussian noise, we see that the flag mean outperforms the competing methods in ARI and F1-measure but falls under Spectrum’s average silhouette scores. This indicates that our method captured the proper clusters accurately but the final estimated clusters were not as “tight” as Spectrum’s. We also see that, in general, we have a lower variance in the case of purely Gaussian data. With microbiome data added, the performance of all methods suffer. We still see the flag mean of subspaces outperforming the others in average ARI and F1-score, but the F1-scores are much more variable. Again, the flag mean has a lower sillhouette score than the competitors. In general, these outcomes are consistent with what we see across simulation settings. We outperform our competitors in accuracy with a higher average and less variable ARI and F1-scores, but the competing methods will outperform the flag mean in silhouette score. See the Supplementary Information for results from all simulation studies.

**Figure 2:**
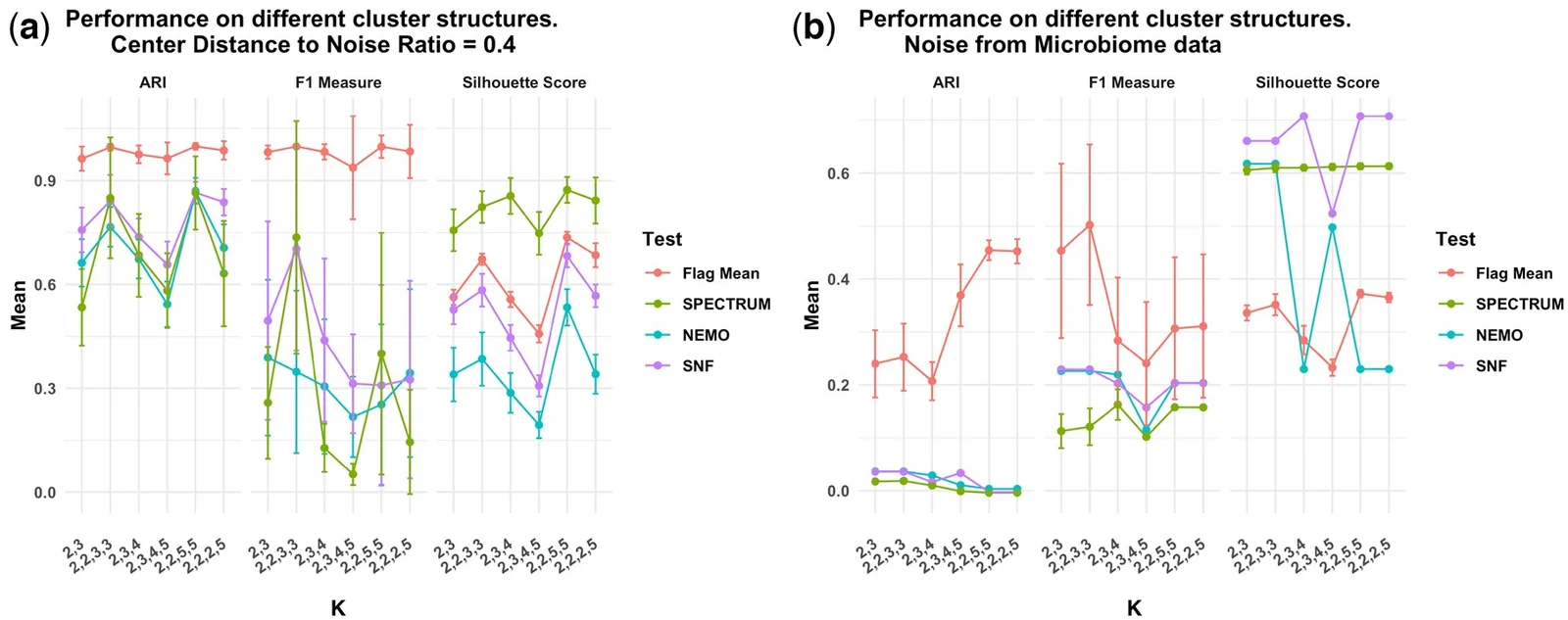
± Results from 1000 simulations with a minimum center distance to noise ratio of 0.4 (a) and CLR transformed microbiome counts added to the underlying MVN cluster structure (b). Points represent averages with error bars showing 1 SD.

In our final simulation study, we see that it is difficult to select the proper number of clusters within the joint clustering space. Most indices never perform well, and indices tended to estimate high numbers of clusters or low numbers of clusters no matter the simulation setting. The inaccuracy in common estimators like the *ccc* index or the *gap* index was surprising. Despite this challenging setting, we found some indices to be reasonably reliable.

Both von Luxburg (2007) and Ng *et al*. (2002) focus on understanding the spectral clustering algorithm through the normalized Laplacian’s eigen-decomposition. Further details can be found in the Supplementary Materials. The methods that seem to be the most reliable for estimating *k* are those that measure the eigenvalues of the within cluster sums of squares and cross products. We believe this is why *traceW* gave the best performance of all the tested methods. Other methods that performed reasonably well were the *hartigan*, *kl*, and *traceCovW* indices. However, the *hartigan* and *kl* indices only did better with more than 2-views and a higher number of groups. In general, the indices that looked for the smallest number of clusters that satisfied some metric, e.g. the *gap* index, did not perform very well. Our simulations had many clusters within it, so this is not the setting for which they were designed. This also means that we should not rely on the mode estimator (“majority rule”) for *k* as is suggested by *NbClust*. We think that the most appropriate indices to use in this setting are those that do not necessarily search for lower numbers of clusters. We see that the *traceW* index performs the best in the scenarios we considered.

### 3.2 Ventilator-associated pneumonia

We applied our proposed method and our competitors to the VAP dataset. The heatmaps of the estimated adjacency matrices from the microbiome and transcriptome data show approximately two and three groups, respectively (Fig. 3a and b). The *traceW* index estimated 4 groups within the joint space. Figure 3c displays the final estimated clusters projected into the first 3 dimensions of the flag mean, μ. A brief summary of the estimated clustering for these data are in Table 1. Spectrum estimated two clusters and SNF gave two estimated clusters (with different assignments than Spectrum), whereas both NEMO and SCM produced nine clusters.

**Figure 3:**
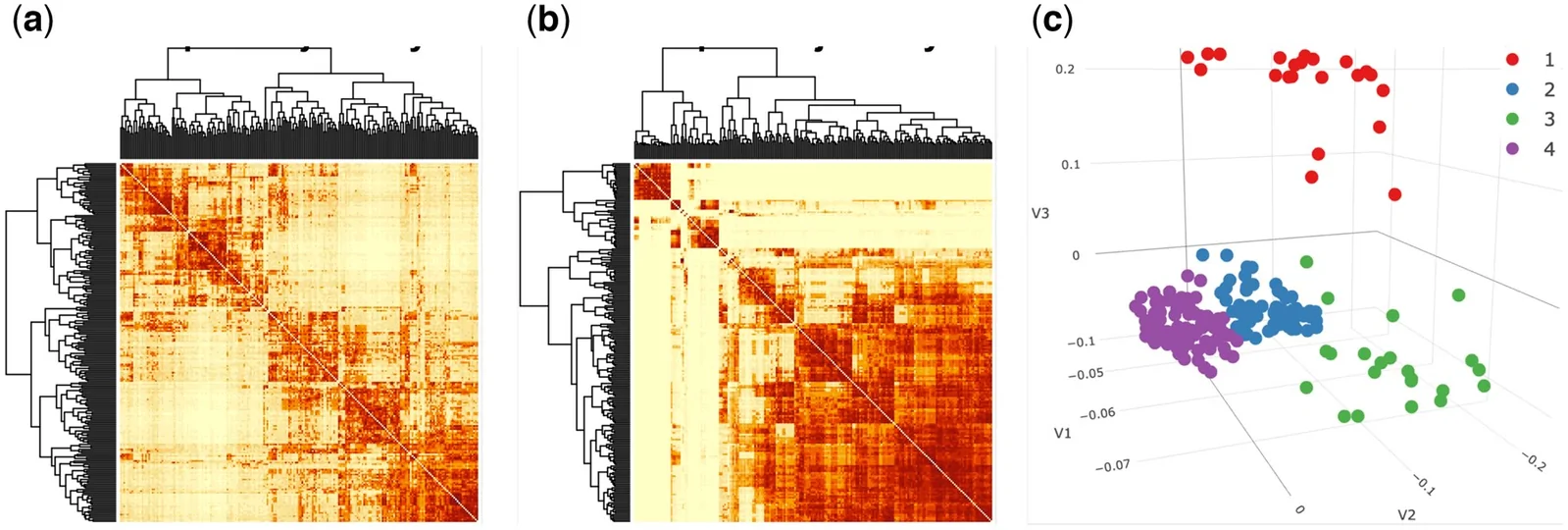
Heatmaps of the adjacency matrices from the microbiome data (a) and transcriptome data (b). The final estimated clusters are displayed within the first three left singular vectors of the flag mean in (c).

**Table 1:** Summary of estimated clusters in the VAP data.

Method	No. of clusters	Silhouette score
**Flag mean**	4	0.626
**Spectrum**	2	0.865
**NEMO**	9	0.150
**SNF**	2	0.565
**SCM**	9	−0.258

### 3.3 TCGA breast cancer application

To assess performance in a clinically annotated cancer cohort, we applied the proposed approach to the TCGA breast cancer benchmark setting, using the breast subtype with multi-omic views derived from gene expression, DNA methylation and miRNA data, together with PAM50 and survival data. The analysis pipeline kept primary tumor samples, aligned patients across views, and carried forward downstream survival and subtype analyses in the integrated cohort. The survival endpoints used were OS and PFI.

Across methods, the inferred cluster granularity varied substantially. The Flag mean methodology selected six clusters; SCM was then fit with K=6 for comparability. Meanwhile, Spectrum and SNF each returned two clusters and NEMO returned three. In this cohort, agreement with PAM50 did not track with prognostic utility. SNF, Spectrum and NEMO showed higher adjusted Rand indices (ARI) with PAM50 than the flag mean, but the flag mean achieved the strongest separation for PFI among all methods (log-rank *P* = .015), while the competing methods showed weaker PFI signal (SCM *P* = .0790, Spectrum *P* = .124, SNF *P* = .0606, NEMO *P* = .0758). This ranking was also evident in the PFI Kaplan–Meier curves, where the flag mean solution showed clearer survival separation across its six clusters than the comparator solutions, with the poorest trajectories arising from clusters 3 and 4. By contrast, NEMO gave the strongest OS signal (*P* = .0248), whereas flag mean was not the best performer for OS. Together, these results suggest that the flag mean solution captures prognostic structure that is not fully summarized by concordance with canonical intrinsic subtype labels alone, while acknowledging that its OS separation was not significant. (Table 2 and Fig. 4a).

**Figure 4:**
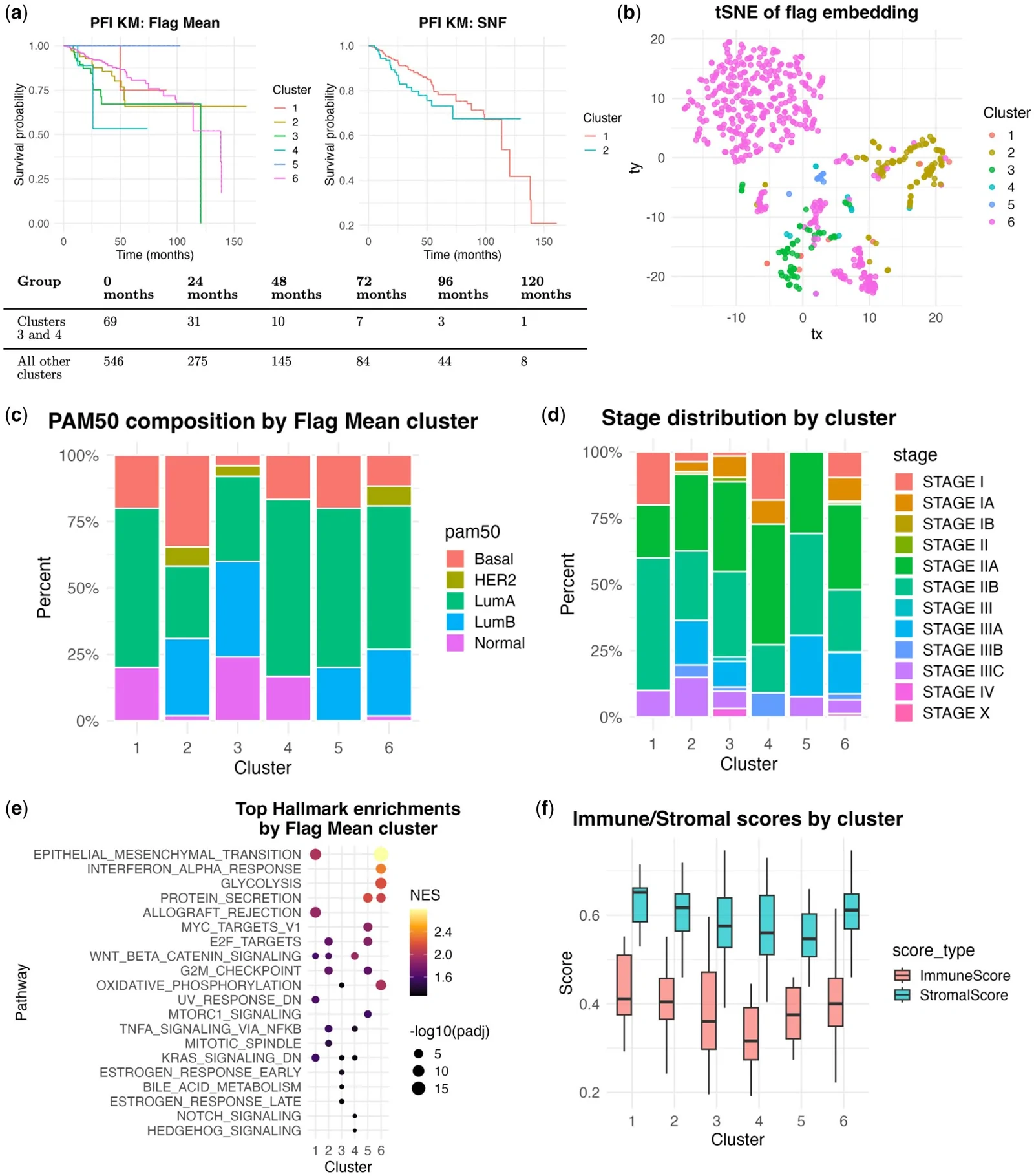
Biological and clinical characterization of flag mean clusters in TCGA breast cancer. (a) Kaplan-Meier curves for PFI by flag mean cluster and SNF cluster. flag mean identifies a subset of clusters with visibly poorer PFI, whereas SNF yields weaker prognostic separation. The number-at-risk table below the Kaplan-Meier panel gives the patients at risk at 0, 24, 48, 72, 96, and 120 months for clusters 3 and 4 versus all other clusters. (b) Two-dimensional t-SNE projection of the same embedding, colored by flag mean cluster. (c) PAM50 subtype composition within each flag mean cluster, shown as within-cluster percentages. Together, these panels show that the flag mean solution yields a finer six-cluster stratification with visible structure in the learned embedding and only partial alignment with PAM50 subtypes. (d) Stacked bar plot showing the proportion of tumors in each recorded pathologic stage category within each of the six flag mean clusters. (e) Top Hallmark pathway enrichments for each flag mean cluster. Points are colored by normalized enrichment score and scaled by enrichment significance, measured as $-{\;\text{log}\;}_{10}(\text{adjusted}\;p)$. Distinct clusters show different pathway programs, including epithelial-mesenchymal transition, interferon response, glycolysis, cell-cycle associated signatures and estrogen-response programs. (f) Immune score and stromal score distributions across flag mean clusters, showing cluster-level differences in tumor microenvironment composition.

**Table 2:** Comparison of clustering methods in the TCGA breast cancer data.^a^

Method	ARI	OS *p*	PFI *p*	No. of clusters
**Flag mean**	0.0943	0.164	0.0150	6
**Spectrum**	0.256	0.254	0.124	2
**SNF**	0.314	0.231	0.0606	2
**NEMO**	0.279	0.0248	0.0758	3
**SCM**	0.0240	0.601	0.0790	6

a Agreement with PAM50 is measured by adjusted Rand index, and survival separation is assessed by log-rank tests for OS and PFI.

The six cluster flag mean solution also produced a more structured partition than the competing methods. The embedding visualizations were consistent with this pattern: the t-SNE projection showed several well-defined regions in the learned flag embedding, including a large, well-separated cluster and smaller localized groups, rather than a single dominant continuum. The PAM50 composition plot further showed that these clusters were not a simple relabeling of the intrinsic subtype; instead, several flag mean clusters contained mixed PAM50 composition, supporting the idea that the joint embedding captures biology that is only partially aligned with standard subtype labels. The stage distribution plot showed additional clinical heterogeneity across the flag mean clusters, but no cluster was defined by a single stage category. In particular, the poorer-prognosis clusters remained stage-mixed rather than representing only more advanced disease, suggesting that the survival differences were not solely attributable to stage composition (Fig. 4b–d).

Biological characterization of the flag mean clusters supported this interpretation. Hallmark enrichment analysis identified distinct pathway programs across the six clusters, including epithelial–mesenchymal transition and immune-response signatures in some clusters, cell-cycle and MYC/E2F programs in others, and estrogen-response or oxidative phosphorylation programs in additional groups. The tumor microenvironment analysis likewise showed cluster-level differences in immune and stromal scores, indicating that the inferred partition reflects variation in both tumor-intrinsic and microenvironment-associated biology. These patterns are consistent with the intended use of the flag mean framework, which integrates view-specific spectral structure before final clustering rather than enforcing agreement at the graph level (Fig. 4e and f).

To connect this TCGA partition to the flag geometry, we quantified how each flag mean direction drew support from the view subspaces. For flag direction $\mu_{j}$, the view-contribution score was defined as $c_{vj}=\|U_{v}^{⊤}\mu_{j}{\|}_{2}^{2}$, with $\sum_{v}c_{vj}=\sigma_{j}^{2}$ and $f_{vj}=c_{vj}/\sigma_{j}^{2}$ giving the fraction supplied by view *v*. The direction that best separated clusters 3 and 4 from all other patients was the leading direction, $\mu_{1}$ (AUC = 0.90, Cohen’s *d *= 2.18), with similar fractions from expression (0.38), methylation (0.31), and miRNA (0.31). Aggregating over the cluster 3 and 4 separation axis gave methylation 0.44, expression 0.37, and miRNA 0.19, so no single view dominated this axis. Other flag mean directions were more view-specific, including $\mu_{2}$ with methylation fraction 0.03 and $\mu_{6}$ with methylation fraction 0.55, consistent with the interpretation that earlier directions can encode cross-view consensus while later directions retain view-specific structure (Fig. 15, available as supplementary data at *Bioinformatics Advances* online).

### 3.4 Molecular characterization of clusters 3 and 4

To characterize the molecular features associated with the high-risk axis, we contrasted clusters 3 and 4 with the remaining TCGA clusters in feature-wise models. In the full-sample unadjusted analysis, 505 genes, 1306 CpGs, and 13 miRNAs were differentially expressed at an FDR <0.05 The hallmark enrichment of the expression contrast was consistent with a luminal-like and immune-cold profile, with lower epithelial-mesenchymal transition, higher allograft rejection, interferon-alpha and interferon-gamma responses, inflammatory response, upregulation of KRAS signaling, downregulation of UV response, IL6-JAK-STAT3 signaling, TNF-alpha signaling via NF-kappaB, and complement, together with higher estrogen-response programs (Fig. 16, available as supplementary data at *Bioinformatics Advances* online).

Because clusters 3 and 4 were enriched for luminal tumors, we also fit models adjusted for PAM50, stage, nodal status, and age. After adjustment, the remaining expression signal was smaller but retained lower proliferation and metabolism programs, including MTORC1 signaling, E2F targets, G2M checkpoint, MYC targets, and oxidative phosphorylation. Three genes showed significant associations with FDR <0.05 in the adjusted expression model, including lower PRDX1 and higher OVCH2 and C1orf111 in clusters 3 and 4 (Tables 4–6, available as supplementary data at *Bioinformatics Advances* online). We therefore use these analyses to annotate the molecular context of the high-risk axis, while the survival models in Table 3 provide the primary evidence that this axis carries prognostic information beyond subtype and clinical variables.

**Table 3:** Post hoc Cox proportional hazards analyses for PFI using a high-risk indicator defined as flag mean clusters 3 and 4.^a^

Model	Adjustment	HR	*P*-value
**Cox model 1**	PAM50 subtype	3.209	.0027
**Cox model 2**	strata(PAM50 subtype)	2.949	.0062
**Cox model 3**	PAM50 + node + stage + age	2.893	.0104
**LRT**	Add cluster 3/4 to stratified PAM50 model	–	.0127

a HR correspond to the high-risk group versus all other clusters.

A notable feature of the TCGA analysis was the behavior of clusters 3 and 4. In the flag mean PFI Kaplan–Meier curves, these clusters showed the poorest outcomes relative to the remaining groups, motivating a post hoc high-risk definition that combined clusters 3 and 4. Cluster 4 was small (n=10, two PFI events), but the high-risk effect was not carried only by this small cluster: cluster 3 contained 59 patients and 13 PFI events and remained associated with shorter PFI when tested against all other clusters with PAM50 adjustment (HR = 2.80, 95% CI 1.19 to 6.57, *P* = .018; Tables 7 and 8, available as supplementary data at *Bioinformatics Advances* online). This aggregation was not explained by PAM50 composition alone. In Cox models, the high-risk indicator remained associated with shorter PFI after adjustment for collapsed PAM50 subtype (HR = 3.21, *P* = .0027), and the effect persisted in a PAM50-stratified analysis (HR = 2.95, *P* = .0062). Importantly, the signal remained significant after additional clinical adjustment for nodal status, stage and age at diagnosis (HR = 2.89, *P* = .0104). A likelihood ratio test also supported the addition of the high-risk grouping beyond PAM50 stratification alone (*P* = .0127). Thus, although the flag mean showed relatively low agreement with PAM50 by ARI, the cluster 3/4 axis identified a prognostic subgroup with information beyond intrinsic subtype and routine clinical staging variables (Table 3). In the Supplementary Materials, we perform a similar set of analyses with the METABRIC cohort (Supplementary Materials). This aggregation was not explained by PAM50 composition alone. In Cox models, the high-risk indicator remained associated with shorter PFI after adjustment for collapsed PAM50 subtype (HR = 3.21, *P* = .0027), and the effect persisted in a PAM50-stratified analysis (HR = 2.95, *P* = .0062). Importantly, the signal remained significant after additional clinical adjustment for nodal status, stage and age at diagnosis (HR = 2.89, *P* = .0104). A likelihood ratio test also supported the addition of the high-risk grouping beyond PAM50 stratification alone (*P* = .0127). Thus, although the flag mean showed relatively low agreement with PAM50 by ARI, the cluster 3/4 axis identified a prognostic subgroup with information beyond intrinsic subtype and routine clinical staging variables (Table 3). In the Supplementary Materials, we perform a similar set of analyses with the METABRIC cohort (Supplementary Materials).

## 4 Discussion

In this work we focused on unsupervised clustering for multi-omics data. Specifically, we wanted to tackle the issue of different grouping structures across views. Currently, multi-view clustering research focuses on algorithms that assume the same number of clusters in each view and that the members of each cluster are consistent across views. Having different numbers of clusters will obviously violate both of these assumptions. The methods that do not make these assumptions are “consensus clustering” methods (Vega-Pons and Ruiz-Shulcloper 2011), which consolidate clustering labels from each view using various formulas. We aimed to develop a method that was able to integrate clustering information prior to assigning clustering labels in the presence of different numbers of clusters.

Other spectral clustering approaches often aim to combine the estimated graphs by combining the adjacency matrices together. This is the case with SNF and Spectrum. We instead focus on combining the estimated clustering subspaces using the flag mean. The flag mean gave a higher cluster accuracy in almost every setting. Spectrum performed as well as our proposed method in some 2-view studies but lacked consistent performance. Our method had middling silhouette scores, but we argue that the clustering accuracy is more important than the separation.

Selecting the proper number of clusters will always be a difficult challenge in unsupervised clustering. We make this problem more difficult by allowing *k* to change across views. Assuming we have collected the proper eigenvectors from each view, we see that the *traceW* index estimates the proper number of clusters consistently in the settings we explored.

We applied our proposed method and three other well known multi-omics clustering methods to the TCGA and VAP datasets. We saw very different estimated clusters from all methods tested. We also saw evidence of a different number of clusters for each view suggesting that the assumption of the same number of clusters may not be reasonable for these data. We are obviously unable to calculate an ARI or F1-measure, but the silhouette scores from each method reflected our simulation results.

The TCGA high-risk analysis should be interpreted with caution because the flag mean clusters were unbalanced, particularly cluster 4. The survival signal did not appear to be a small-cluster artifact alone: cluster 3 contained 59 patients with 13 PFI events and was individually associated with shorter PFI, the combined cluster 3 and 4 indicator remained associated with PFI after PAM50 and clinical adjustment, and nodal status did not explain the association. In the same patients, the high-risk hazard ratios (HR) was 3.08 without nodal status and 2.69 after adding nodal status, with the association remaining significant (*P* = .013; Table 9, available as supplementary data at *Bioinformatics Advances* online). The molecular feature analyses were also limited by PAM50 coverage, since PAM50 calls were available for 384 of 621 patients and the adjusted high-risk group contained 31 patients. These results support a cautious interpretation of the TCGA cluster 3 and 4 axis and motivate further external validation. The METABRIC analysis in the Supplementary Materials provides such a check and also shows that the strength of the flag mean survival signal may depend on the cohort and omics structure.

Overall, the flag mean is a fast, flexible, and useful tool for combining multiple omics data sources for unsupervised clustering.

## Supplementary Material

vbag217_Supplementary_Data

## References

[vbag217-B1] Argelaguet R , Arnol D, Bredikhin D et al MOFA+: a statistical framework for comprehensive integration of multi-modal single-cell data. Genome Biol 2020;21:111. 10.1186/s13059-020-02015-1 PMC7212577 32393329

[vbag217-B2] Bjorck A , Golub GH. Numerical methods for computing angles between linear subspaces. Math Comput 1973;27:579–94. 10.2307/2005662

[vbag217-B3] Carson T , Mixon DG, Villar S. Manifold optimization for k-means clustering. In: *2017 International Conference on Sampling Theory and Applications (SampTA)*. 2017, 73–7, Tallin, Estonia. 10.1109/SAMPTA.2017.8024388

[vbag217-B4] Charrad M , Ghazzali N, Boiteau V et al NbClust: an R package for determining the relevant number of clusters in a data set. *J Stat Soft* 2014;61:1–36.

[vbag217-B5] Chen C , Ng MK, Zhang S. Block spectral clustering methods for multiple graphs. Numer Linear Algebra App 2017;24:e2075. 10.1002/nla.2075

[vbag217-B6] Chen Y , Wen Y, Xie C et al Mocss: multi-omics data clustering and cancer subtyping via shared and specific representation learning. iScience 2023;26:107378. 10.1016/j.isci.2023.107378 PMC10407241 37559907

[vbag217-B7] Chikhi NF. Multi-view clustering via spectral partitioning and local refinement. Inf Process Manag 2016;52:618–27. 10.1016/j.ipm.2015.12.007

[vbag217-B8] De Sa VR. Spectral clustering with two views. In: *ICML Workshop on Learning with Multiple Views*. Workshop on Learning with Multiple Views, 22nd ICML Bonn, Germany, 2005. 20–7.

[vbag217-B9] Draper B , Kirby M, Marks J et al A flag representation for finite collections of subspaces of mixed dimensions. Linear Algebra Appl 2014;451:15–32. 10.1016/j.laa.2014.03.022

[vbag217-B10] Edelman A , Arias TA, Smith ST. The geometry of algorithms with orthogonality constraints. SIAM J Matrix Anal Appl 1998;20:303–53. 10.1137/S0895479895290954

[vbag217-B11] Gao Q , Xia W, Wan Z et al Tensor-SVD based graph learning for multi-view subspace clustering. AAAI 2020;34:3930–7. 10.1609/aaai.v34i04.5807

[vbag217-B12] Jia Y , Liu H, Hou J et al Multi-view spectral clustering tailored tensor low-rank representation. *IEEE Trans Circuits Syst Video Technol* 2021;31:4784–97.

[vbag217-B13] John CR , Watson D, Barnes MR et al Spectrum: fast density-aware spectral clustering for single and multi-omic data. Bioinformatics 2020;36:1159–66. 10.1093/bioinformatics/btz704 PMC7703791 31501851

[vbag217-B14] Khan A , Maji P. Approximate graph Laplacians for multimodal data clustering. IEEE Trans Pattern Anal Mach Intell 2021a;43:798–813. 10.1109/TPAMI.2019.2945574 31603770

[vbag217-B15] Khan A , Maji P. Multi-Manifold optimization for multi-view subspace clustering. IEEE Trans Neural Netw Learn Syst 2021b;33:3895–907. 10.1109/TNNLS.2021.3054789 33606638

[vbag217-B16] Knyazev AV , Argentati ME. Principal angles between subspaces in an a-based scalar product: algorithms and perturbation estimates. SIAM J Sci Comput 2002;23:2008–40. 10.1137/S1064827500377332

[vbag217-B17] Kumar A , Rai P, Daume H. Co-regularized multi-view spectral clustering. In: Shawe-Taylor J, Zemel R, Bartlett P, et al (eds), *Advances in Neural Information Processing Systems*, Vol 24. Red Hook, NY: Curran Associates, Inc., 2011. https://papers.nips.cc/paper/2011/hash/31839b036f63806cba3f47b93af8ccb5-Abstract.html

[vbag217-B18] Lock EF , Dunson DB. Bayesian consensus clustering. Bioinformatics 2013;29:2610–6. 10.1093/bioinformatics/btt425 PMC3789539 23990412

[vbag217-B19] Love MI , Huber W, Anders S. Moderated estimation of fold change and dispersion for rna-seq data with deseq2. Genome Biol 2014;15:550. 10.1186/s13059-014-0550-8 PMC4302049 25516281

[vbag217-B20] Luo C , Xu J, Ren Y et al Simple contrastive multi-view clustering with data-level fusion. In: *Proceedings of the Thirty-Third International Joint Conference on Artificial Intelligence*, Vol. 5. Darmstadt: International Joint Conferences on Artificial Intelligence (IJCAI), 2024.

[vbag217-B21] Luo S , Zhang C, Zhang W et al Consistent and specific multi-view subspace clustering. Proc AAAI Conf Artif Intell 2018;32:3730–7. 10.1609/aaai.v32i1.11617

[vbag217-B22] Luo W , Li B. Combining eigenvalues and variation of eigenvectors for order determination. Biometrika 2016;103:875–87. 10.1093/biomet/asw051

[vbag217-B23] Maimon O , Rokach L. *Data Mining and Knowledge Discovery Handbook*. New York: Springer Science & Business Media, 2006.

[vbag217-B24] Mick E , Tsitsiklis A, Kamm J et al Integrated host/microbe metagenomics enables accurate lower respiratory tract infection diagnosis in critically ill children. J Clin Invest 2023;133:1–13. 10.1172/JCI165904 PMC10065066 37009900

[vbag217-B25] Mitra S , Saha S. A multiobjective multi-view cluster ensemble technique: application in patient subclassification. PLoS One 2019;14:e0216904. 10.1371/journal.pone.0216904 PMC6533037 31120942

[vbag217-B26] Mitra S , Saha S, Hasanuzzaman M. Multi-view clustering for multi-omics data using unified embedding. Sci Rep 2020;10:13654. 10.1038/s41598-020-70229-1 PMC7423957 32788601

[vbag217-B27] Monk D. The geometry of flag manifolds. Proc Lond Math Soc 1959;s3-9:253–86. 10.1112/plms/s3-9.2.253

[vbag217-B28] Mourani PM , Sontag MK, Williamson KM, et al Eunice Kennedy Shriver National Institute of Child Health and Human Development Collaborative Pediatric Critical Care Research Network (CPCCRN). Temporal airway microbiome changes related to ventilator-associated pneumonia in children. Eur Respir J 2021;57:2001829. 10.1183/13993003.01829-2020 PMC7979474 33008935

[vbag217-B29] Nepusz T , Sasidharan R, Paccanaro A. SCPS: a fast implementation of a spectral method for detecting protein families on a genome-wide scale. BMC Bioinformatics 2010;11:120. 10.1186/1471-2105-11-120 PMC2841596 20214776

[vbag217-B30] Ng A , Jordan M, Weiss Y. On spectral clustering: analysis and an algorithm. In: *Advances in Neural Information Processing Systems*, Vol. 14. Vancouver, BC: MIT Press, 2002. https://papers.nips.cc/paper/2001/hash/801272ee79cfde7fa5960571fee36b9b-Abstract.html

[vbag217-B31] Nguyen H , Shrestha S, Draghici S et al PINSPlus: a tool for tumor subtype discovery in integrated genomic data. Bioinformatics 2019;35:2843–6. 10.1093/bioinformatics/bty1049 30590381

[vbag217-B32] Nguyen T , Tagett R, Diaz D et al A novel approach for data integration and disease subtyping. Genome Res 2017;27:2025–39. 10.1101/gr.215129.116 PMC5741060 29066617

[vbag217-B33] Pennec X. Barycentric subspace analysis on manifolds. Annals Stat 2018;46:2711–46. 10.1214/17-AOS1636-AOS1636

[vbag217-B34] R Core Team. R: A Language and Environment for Statistical Computing. Vienna, Austria: R Foundation for Statistical Computing, 2022. https://www.R-project.org/

[vbag217-B35] Rappoport N , Shamir R. Multi-omic and multi-view clustering algorithms: review and cancer benchmark. Nucleic Acids Res 2018;46:10546–62. 10.1093/nar/gky889 PMC6237755 30295871

[vbag217-B36] Rappoport N , Shamir R. NEMO: cancer subtyping by integration of partial multi-omic data. Bioinformatics 2019;35:3348–56. 10.1093/bioinformatics/btz058 PMC6748715 30698637

[vbag217-B37] Rohart F , Gautier B, Singh A et al mixOmics: an R package for ‘omics feature selection and multiple data integration. PLoS Comput Biol 2017;13:e1005752. 10.1371/journal.pcbi.1005752 PMC5687754 29099853

[vbag217-B38] Shen R , Olshen AB, Ladanyi M. Integrative clustering of multiple genomic data types using a joint latent variable model with application to breast and lung cancer subtype analysis. Bioinformatics 2009;25:2906–12. 10.1093/bioinformatics/btp543 PMC2800366 19759197

[vbag217-B39] Speicher NK , Pfeifer N. Integrating different data types by regularized unsupervised multiple kernel learning with application to cancer subtype discovery. Bioinformatics 2015;31:i268–i275. 10.1093/bioinformatics/btv244 PMC4765854 26072491

[vbag217-B40] Vega-Pons S , Ruiz-Shulcloper J. A survey of clustering ensemble algorithms. Int J Patt Recogn Artif Intell 2011;25:337–72. 10.1142/S0218001411008683

[vbag217-B41] von Luxburg U. A tutorial on spectral clustering. Stat Comput 2007;17:395–416. 10.1007/s11222-007-9033-z-007-9033-z

[vbag217-B42] Wang B , Mezlini AM, Demir F et al Similarity network fusion for aggregating data types on a genomic scale. Nat Methods 2014;11:333–7. 10.1038/nmeth.2810 24464287

[vbag217-B43] Wang B , Zeng C, Chen M et al Towards learnable anchor for deep multi-view clustering. Proc AAAI Conf Artif Intell 2025;39:21044–52. 10.1609/aaai.v39i20.35401

[vbag217-B44] Wang Y , Jiang Y, Wu Y et al Spectral clustering on multiple manifolds. IEEE Trans Neural Netw 2011;22:1149–61. 10.1109/TNN.2011.2147798 21690009

[vbag217-B45] Wei L , Zeng W, Wang H. K-means clustering with manifold. In: *2010 Seventh International Conference on Fuzzy Systems and Knowledge Discovery*, Vol. 5. Yantai, Shandong Province, 2010, 2095–9. 10.1109/FSKD.2010.5569712

[vbag217-B46] Xia R , Pan Y, Du L et al Robust Multi-View spectral clustering via low-rank and sparse decomposition. Proc AAAI Conf Artif Intell 2014;28:2149–55. 10.1609/aaai.v28i1.8950

[vbag217-B47] Xu J , Ren Y, Tang H et al Multi-vae: learning disentangled view-common and view-peculiar visual representations for multi-view clustering. In: *Proceedings of the IEEE/CVF International Conference on Computer Vision.* Montreal. 2021, 9234–43.

[vbag217-B48] Yao S , Yu G, Wang J et al Multi-view multiple clustering. arXiv, arXiv:1905.05053, 2019, preprint: not peer reviewed.

[vbag217-B49] Ye K , Wong KS-W, Lim L-H. Optimization on flag manifolds. *Math Program* 2022;194:621–60.

[vbag217-B50] Yu Y , Zhang L-H, Zhang S. Simultaneous clustering of multiview biomedical data using manifold optimization. Bioinformatics 2019;35:4029–37. 10.1093/bioinformatics/btz217 30918942

[vbag217-B51] Zhang C , Fu H, Hu Q et al Generalized latent multi-view subspace clustering. IEEE Trans Pattern Anal Mach Intell 2020;42:86–99. 10.1109/TPAMI.2018.2877660 30369436

[vbag217-B52] Zhang S , Zhao H, Ng MK. Functional module analysis for gene coexpression networks with network integration. IEEE/ACM Trans Comput Biol Bioinform 2015;12:1146–60. 10.1109/TCBB.2015.2396073 PMC4664071 26451826

[vbag217-B53] Zhang X , Zhou Z, Xu H et al Integrative clustering methods for multi-omics data. Wiley Interdiscip Rev Comput Stat 2022;14:e1553. 10.1002/wics.1553 PMC9097984 35573155

[vbag217-B54] Zhang Y , Yang W, Liu B et al Multi-view spectral clustering via tensor-SVD decomposition. In: *2017 IEEE 29th International Conference on Tools with Artificial Intelligence (ICTAI)*. Boston, MA, 2017, 493–7. 10.1109/ICTAI.2017.00081

[vbag217-B55] Zhou D , Burges CJ. Spectral clustering and transductive learning with multiple views. In: *Proceedings of the 24th International Conference on Machine Learning*. Corvallis, Oregon: ACM, 2007, 1159–66.

[vbag217-B56] Zong L , Zhang X, Zhao L et al Multi-view clustering via multi-manifold regularized non-negative matrix factorization. Neural Netw 2017;88:74–89. 10.1016/j.neunet.2017.02.003 28214692

