# Supplementary material for: Multi-omic spectral clustering with the flag mean
Source: Bioinform Adv. 2026 Aug 4;6(1):vbag217. doi: 10.1093/bioadv/vbag217 (PMC13692795; doi:10.1093/bioadv/vbag217)
Supplement: Supplementary file 1 [file vbag217_supplementary_data.pdf]

Competing Methods

Here we provide brief overviews of the competing methods. All three of these methods are popular algorithms for multi-omic spectral clustering, all of them assume a common number of clusters across views, and all of them focus on combining graphs from each view prior to clustering.

Similarity Network Fusion

Similarity Network Fusion (SNF) (Wang et al., 2014) has been the gold standard for multi-omics spectral clustering for some time. Given an adjacency matrix A, SNF first normalizes A and creates a k-nearest neighbor adjacency matrix A_*. It next completes multiple graph diffusion iterations $Q_t = A_* Q_{t-1} A_*^T$ with $Q_1 = A_*$. Graph diffusions are a common tool in identifying community structures using graphs. It repeats these steps for each view in the study and combines each Q_t using a simple average $Q_* = \frac{1}{V}\sum_v Q_t^v$. Finally, they perform the spectral clustering algorithm with Q_* as their adjacency matrix.

Spectrum

Spectrum (John et al., 2020) first introduces an 'adaptive density-aware kernel' that takes into account both local and global network structures. We found this to be very useful and we use it in our analysis of the ventilator associated pneumonia data. Their algorithm is very similar to SNF. Given an adjacency matrix A, Spectrum first creates a k-nearest neighbor graph (default is 10), then row normalizes A, then performs several (default is 5) graph diffusion iterations $Q_t = AQ_{t-1}A^T + I$, and finally performs spectral clustering with $Q_t = A^*$ as the adjacency matrix. Here $Q_1 = A$ and I is the identity matrix. In the presence of multi-view data it simply sums the adjacency matrices before completing these steps, whereas SNF completes these steps for each view and then combines them.

NEMO

NEighborhood based Multi-Omics clustering (NEMO) (Rappoport and Shamir, 2019) was developed for cancer subtyping in multi-omics studies. For each adjacency matrix they calculate the 'relative similarity' between the neighboring points. For subjects i and j they measure the similarity between i and j relative to i's k nearest neighbors and to j's k nearest neighbors. The main goal of this is transforming the adjacency matrices to be more comparable across views, since most omics data do not share underlying distributions. They call these adjacency matrices, A_{rs}, 'relative similarity' adjacency matrices. Finally, they take the average of all the 'relative similarity' adjacency matrices, $A^* = \frac{1}{V}\sum_v A_{rs}^v$, and use this as the adjacency matrix for spectral clustering.

Simple Contrastive Multi-View Clustering

Simple Contrastive Multi-View Clustering (SCM) (Luo et al., 2024) is a deep multi-view clustering method that fuses information at the data level. Given multi-view data ${X^v}_{v=1}^V$, SCM first applies min–max normalization within each view and concatenates the results to form a single fused matrix $\bar{X} = [N(X^1), N(X^2), \ldots, N(X^V)]$. It then generates two augmented versions of $\bar{X}$: a noise augmentation that perturbs a random subset of view blocks and a missing-view augmentation that masks out random view blocks to simulate unavailable assays. Both augmented matrices are passed through a shared encoder to produce embeddings $\bar{Z}^n$ and $\bar{Z}^m$, and SCM uses an InfoNCE contrastive objective to pull together embeddings from the same sample across augmentations while pushing apart embeddings from different samples. Finally, clustering is performed on the learned representation (e.g., by applying k-means to the learned features).

Simulation Studies

Changing number of features across views

In the more basic simulations in the main article, the number of groups changed but the number of features across views and amount of noise within each view were the same. We conduct this simulation study to see if the number of features impacts the performance of the methods. We investigate 2, 3, and 4 view studies. The number of clusters in each type is $k = [2,3], k = [2,3,4]$, and $k = [2,3,4,5]$, respectively. These simulations were performed with $\tau = 5$ for all views.

Changing noise across views

We then investigate if changing the amount of noise within each view has an impact. The first simulation changed the amount of noise, τ, but τ stayed constant across all views within a single setting. Here we change the amount of noise among the views. We keep the same clustering set up as the previous simulation study, i.e., $k = [2,3], k = [2,3,4]$, and $k = [2,3,4,5]$ for 2, 3, and 4 view studies, respectively. We set $p = 60$ for all simulations again.

Methods for estimating the number of clusters within the joint space

We evaluate a total of 31 different indices for cluster estimation. For full details on most of these methods see (Charrad et al., 2012) and the references within. The Ball index is similar to the objective function for k-means clustering (Ball and Hall, 1965). It calculates

$$ball = \frac{W}{k},$$

where W is the within clusters sums of squares for a given number of clusters, k. The largest difference between different settings of k is taken as the optimal solution. The Beale index (Beale, 1969) is defined as

$$beale = \frac{\frac{W_m - W_k + W_l}{W_k + W_l}}{\left(\frac{n_m - 1}{n_m - 2}\right) 2^{2/p} - 1},$$

where $W_q = \sum_{k=1}^{q} \sum_{i \in C_k} (x_i - c_k)(x_i - c_k)^T$ is the within-group dispersion matrix for data clustered into q clusters, x_i is the p dimensional vector of observation i in cluster k, and c_k is the centroid of cluster C_k. It is assumed that the clusters C_k and C_l merge to form cluster C_m. The optimal numbers of clusters is found by taking *beale* as an $F_{p,(N_m-2)p}$ statistic. It can therefore be used as a test for the presence of clusters, but the maximum is used for the index estimate of k. The cubic clustering criterion (CCC) (Milligan and Cooper, 1985) is defined as

$$ccc = ln\left[\frac{1 - E(R^r)}{1 - R^2}\right] \frac{\sqrt{np/2}}{[0.001 + E(R^r)]^{1.2}},$$

where

$$R^2 = 1 - \frac{trace(W)}{trace(T)}.$$

Here, if X is the $n \times p$ matrix of clustering data $T = X^T X$ is the $p \times p$ total sums of squares and crossproducts (SSCP) matrix, Z is a cluster indicator matrix with element $z_{ik} = 1$ if the ith observation is in cluster C_k and 0 otherwise, $\tilde{X} = (Z^T Z)^{-1} Z^T X$, $B = \tilde{X}^T Z^T Z \tilde{X}$, and $W = T - B$. The maximum of *ccc* across the number of clusters is used to indicate the optimal number of clusters. The CH index (Caliński and Harabasz, 1974) aims to maximize the function

$$CH = \frac{trace(B_q)/(q-1)}{trace(W_q)/(n-q)},$$

where $W_q = \sum_{k=1}^{q} \sum_{i \in C_k} (x_i - c_k)(x_i - c_k)^T$ is the within-group dispersion matrix for data clustered into q clusters, $B_q = \sum_{k=1}^{q} n_k (c_k - c)(c_k - c)^T$ is the between-group dispersion matrix for q clusters, x_i is the p dimensional vector of observation i in cluster k, and c_k is the centroid of cluster C_k, c is the centroid of the data, and n_k is the number of objects in cluster C_k. The Cindex (Hubert and Baker, 1976) aims to minimize

$$cindex = \frac{Du - rD_{min}}{rD_{max} - rD_{min}},$$

where r is the number of within cluster dissimilarities, Du is the sum of all within cluster dissimilarities, and D_{min} and D_{max} are the minimum and maximum within cluster dissimilarities, respectively. The Davies and Bouldin index (DB) is a function of the ratio of the sum of within-cluster spread to between cluster distance, similar to the silhouette score. The index minimizes

$$DB = 1/k \sum_{q=1}^{k} max_{s,q \neq s} \frac{S_q + S_s}{d_{qs}},$$

where $s, q = 1, ..., k$ are the cluster indicators, $k \geq 2$ is the number of clusters, $d_{qs} = \sqrt{\sum_{j=1}^{p} (c_{qj} - c_{sj})^2}$ is the euclidean distance between the centroids of clusters C_q and C_s, and S_r is the dispersion measure of cluster C_r. The Duda index (Milligan and Cooper, 1985)

$$duda = \frac{W_k + W_l}{W_m}$$

aims to find the smallest number of clusters such that $duda \geq 1 - \frac{2}{\pi p} - 3.2\sqrt{\frac{2\left(1 - \frac{8}{\pi^2 p}\right)}{n_m p}}$. The right hand side of this inequality will be referred to as the *dudaValue*. W_k, W_l, and W_k are as defined above in the CH index. The Dunn index (Dunn†, 1974) is defined as

$$dunn = \frac{min_{1 \leq i < j \leq k} d(C_i, C_j)}{max_{1 \leq r \leq k} diam(C_k)}.$$

$d(C_i, C_j) = min_{x \in C_i, y \in C_j} d(x, y)$ is the dissimilarity function between two clusters C_i and C_j, $diam(C) = max_{x,y \in C} d(x, y)$, and $d(x, y)$ is the euclidean distance. The Frey index (Frey and van Groenewoud, 1972)

$$frey = \frac{d_{v_{j+1}} - d_{v_j}}{d_{s_{j+1}} - d_{v_j}}$$

is a hierarchical algorithm that compares the clustering density between two hierarchy levels (j and $j+1$). The algorithm finds the number of clusters $k*$ at which the ratio stays below 1 for all $k > k*$. d_v and d_s are the outer and inner cluster distances, respectively. The Friedman index (Friedman and Rubin, 1967)

$$friedman = trace(W^{-1}B)$$

is a relatively simple index that aims to find the maximum difference between the index values among different numbers of clusters. W and B is as defined in the *ccc* index. The Gamma index (Milligan and Cooper, 1985) is another ratio index that aims to maximize the

ratio

$$gamma = \frac{s(+) - s(-)}{s(+) + s(-)},$$

where $s(+)$ is the number of concordant comparisons and $s(-)$ is the number of discordant comparisons. The Gap index is a somewhat famous index (Tibshirani et al., 2001). The gap statistic

$$Gap = \frac{1}{B}\sum_{b=1}^{B} logW_{kb} - logW_k,$$

where B is the number of reference data sets generated using uniform prescription, W_{kb} is the within dispersion matrix defined above. The optimal number of clusters is chosen by finding the smallest k such that $Gap(k) \geq Gap(k+1) - s_{q+1}$, where $s_q = \sqrt{\frac{1}{B}\sum_{b=1}^{B}(logW_{kb} - \frac{1}{B}\sum_{b=1}^{B} logW_{kb})^2}$. The Hartigan index (Hartigan, 1975) is another index that aims to maximize its objective function

$$hartigan = \left(\frac{trace(W_k)}{trace(W_{k+1})} - 1\right)(n - k - 1),$$

where W_k is the within group dispersion as defined above. The KL index (Krzanowski and Lai, 1988) aims to maximize its index

$$KL = \left|\frac{DIFF_k}{DIFF_{k+1}}\right|,$$

where $DIFF_k = (k-1)^{2/p} trace(W_{k-1}) - k^{2/p} trace(W_k)$ and W_k is the dispersion as defined above. The Marriot index (Marriott, 1971) is a relatively simple index,

$$marriot = k^2|W|,$$

where W is the within group dispersion matrix as defined above. The maximum difference in the index between different ks determines the estimated number of groups. The McClain index (Milligan and Cooper, 1985) is another method that tried to minimize it's index

$$mcclain = \frac{mean(\sum_{k}^{q=1}\sum_{i=i}^{n_q}\sum_{j=i+1}^{n_q} d_{ij})}{mean(\sum_{q=1}^{k}\sum_{i\in C_q}\sum_{l=q+1}^{k}\sum_{j\in C_l} d_{ij})},$$

where k is the number of clusters, n_k is the number of objects in the qth cluster, and d_{ij} is the distance between the ith and j^{th} objects. The Pseudot index (Milligan and Cooper, 1985) is another method from Duda. It looks for the smallest k such that

$$\frac{B_{kl}}{\frac{W_k + W_l}{n_k + n_l - 2}} \leq \left(\frac{1 - dudaValue}{dudaValue}\right)(n_k + n_l - 2),$$

where B_{kl} and W_j are as described above, and n_j are the number of objects within the clusters. The PtBiserial index (Milligan and Cooper, 1985) looks for the k that maximizes it's index

$$ptbiserial = \frac{(\bar{d}_b - \bar{d}_w)\sqrt{f_w f_b / n_d^2}}{S_d},$$

where $\bar{d}_w$ and $\bar{d}_b$ are average within and between cluster distances, respectively, n_d is the total number of distances, f_w and f_b are the number of within and between cluster distances, respectively, and S_d is the standard deviation of all distances. The Ratkowsky index (Ratkowsky and Lance, 1978) is altered from the original in the *NbClust* package. The package assumes that the true number of groups is constant (as do we) and reduces the index to

$$ratkowsky = mean(\sqrt{B/T}),$$

where B is the sum of squares between clusters and T is the total sums of squares. The Rubin index is similar to many we've discussed in that it aims to minimize

$$rubin = |T|/|W|$$

, where T and W are as defined in other indices like *ccc*. The Scott index

$$scott = nlog(|T|/|W|)$$

is very similar to Rubin and others (Scott and Symons, 1971). T and W are, again, the same matrices as defined in *ccc*, and the algorithm looks for the maximum difference between hierarchy levels as the estimated number of k. The SDbw is an index that looks for partitions

that balance the separation and density of the clusters (Halkidi et al., 2001). The index is defined as

$$sdbw = sep(k) + density.bw(k),$$

where $sep(k)$ is a function that measures the average compactness of a cluster (i.e. the average intra-cluster distance),

$$density.bw(k) = \frac{1}{k(k-1)} \sum_{i} = 1^{q} \left(\sum_{j=1, i \neq j}^{q} \frac{density(u_{ij})}{max(density(c_i), density(c_j))} \right),$$

$density(u) = \sum_{l=1}^{n_{ij}} f(x_l, u)$, $f(x, u)$ is 1 is $d(x, u) \leq stdev$. Here $stdev = \frac{1}{k}\sqrt{\sum_{i=1}^{k} ||\sigma(c_i)||}$ is the average within cluster standard deviation. The Silhouette index is a well known index that measures the average between and within cluster similarities (Kaufman and Rousseeuw, 1990). The index

$$silhouette = \sum_{i=1}^{n} S(i)/n,$$

where $S(i) = \frac{b(i)-a(i)}{max(a(i),b(i))}$ is the silhouette score of subject i, $a(i) = \frac{\sum_{j \in C_r} d_{ij}}{n_r - 1}$ is the average dissimilarity of the ith object to all subjects within cluster C_r, $b(i) = min_{s \neq r} d_{iCs}$, $d_{iCs} = \frac{\sum_{j \in C_s} d_{ij}}{n_s}$ is the average dissimilarity of the ith object to all objects of cluster C_s, and n_r and n_s are the number of subjects with their respective clusters. The Tau index (Milligan and Cooper, 1985) is similar to the *gamma* index. The index

$$tau = \frac{s(+) - s(-)}{\sqrt{(n_d(n_d-1)/2 - t)(n_d(n_d-1)/2)}}$$

has the same numerator as *gamma* but a set denominator with n_d the total number of distances (subjects) and t the number of comparisons of two pairs of points where both pairs represent within cluster comparisons or both pairs are between cluster comparisons. The final two from the *NbClust* package are the TraceW and TraceCovW indices (Friedman and Rubin, 1967; Milligan and Cooper, 1985). Both are relatively simple objective functions with

$$traceW = trace(W)$$

and

$$traceCovW = trace(cov(W)),$$

where W is as defined with the *ccc* index and $cov(\cdot)$ is the covariance matrix. TraceW takes note that its objective function increases monotonically with solutions containing fewer clusters and finds the maximum of the second differences scores. The TraceCovW indices finds the maximum differences scores between levels to indicate the optimal solution.

We also include some extra indices to explore that are not offered by the *NbClust* package. The Ladle estimator aims to find the rank of a matrix by investigating the variation of the eigenvectors. The order of the graph Laplacian corresponds to the number of clusters in this context, and we use the estimated order of the flag mean as the method's estimate of k. We explore two methods of dimension reduction via the Ladle estimator, principal component analysis (LadlePCA) and independent component analysis (LadleICA). The slope statistic (Fujita et al., 2014) calculates the silhouette score $S(k)$ for different ks, and estimates k as $argmax_k = -[S(k+1) - S(k)]S(k)^p$. We leave p as 1 for our study. We also explore the simple 'spectral gap' index as the largest difference in singular values of the flag mean, respectively. Finally, we include the 'mode' estimator. It is simply the most common estimate of k from all indices (the mode). This is sometimes referred to as the "majority rule."

Simulation results

Basic simulations

Here, we summarize the simulation results described but not shown in the main article. The following are the results from changing the τ parameter

When the center distance to noise ratio is 1, i.e. $\tau = 2$, we see relatively similar performances among the methods (Figure S1). NEMO and SNF do not perform as well as the others in most scenarios. Spectrum has as similar ARI and F1-measure to our methods when there are only 2 different cluster structures. Our methods outperform the others in ARI and F1-measure when more that 2 different clusters are present.

When the center distance to noise ratio is 0.286, i.e. $\tau = 7$, we see relatively similar performances among the methods (Figure S2). We see very similar results to when $\tau = 5$, only we see larger standard deviations from all methods. Spectrum, NEMO, and SNF do not perform as well as the proposed methods in ARI and F1-measure in any simulation. Again, Spectrum has the best silhouettes scores.

When the center distance to noise ratio is 0.2, i.e. $\tau = 10$, we see the performance of all methods degrade (Figure S3). Our proposed methods still outperform the others in F1-measure, but there is so much variation that performance is not reliably better than SNF. All methods have a similar ARI. Even though Spectrum performs the worst on ARI and F1-measure, they still maintain an excellent silhouette score.

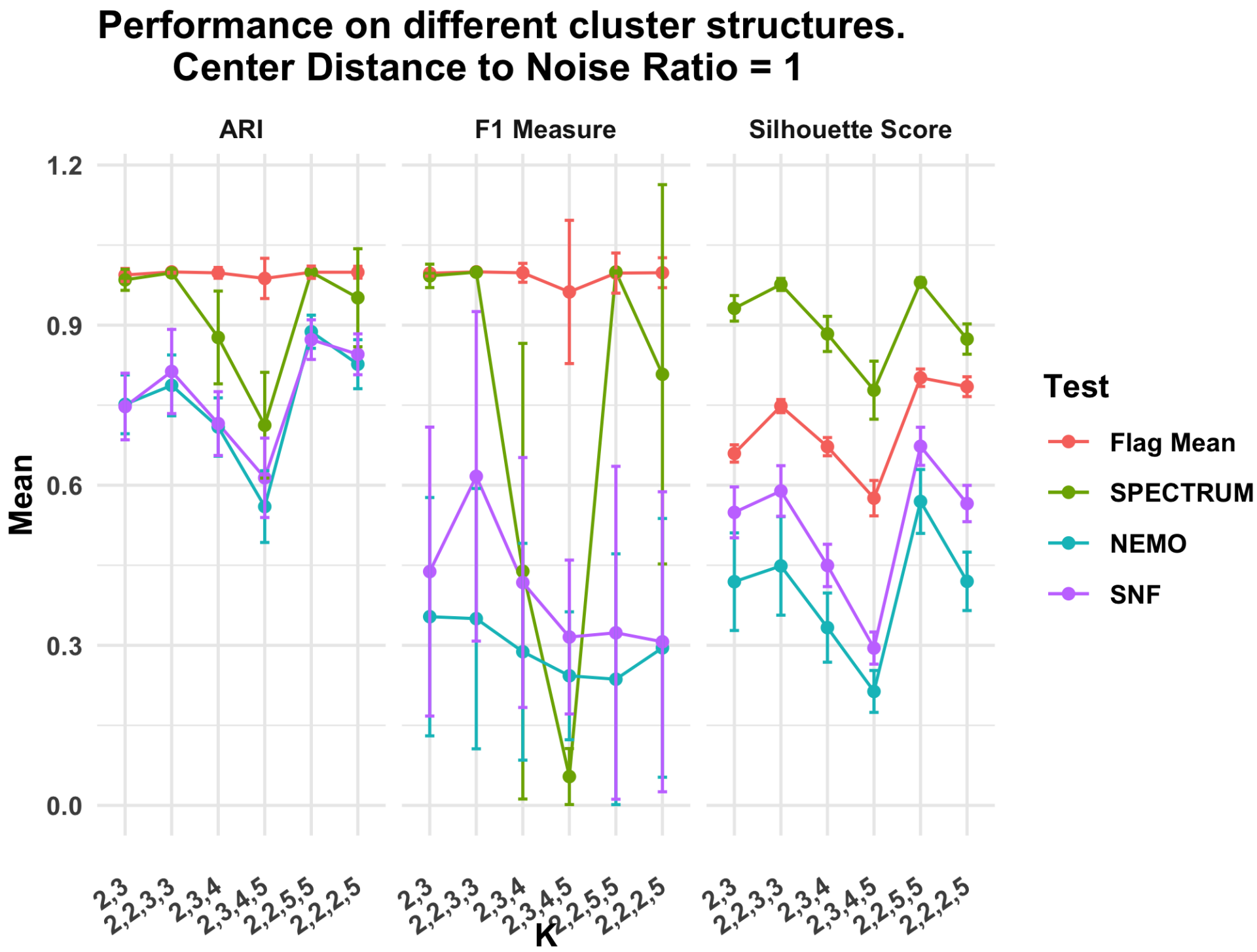

Fig. S1: Results from 5000 simulations with a minimum center distance to noise ratio of 1 ($\tau = 2$). Averages are displayed as dots with error bars showing ± one standard deviation.

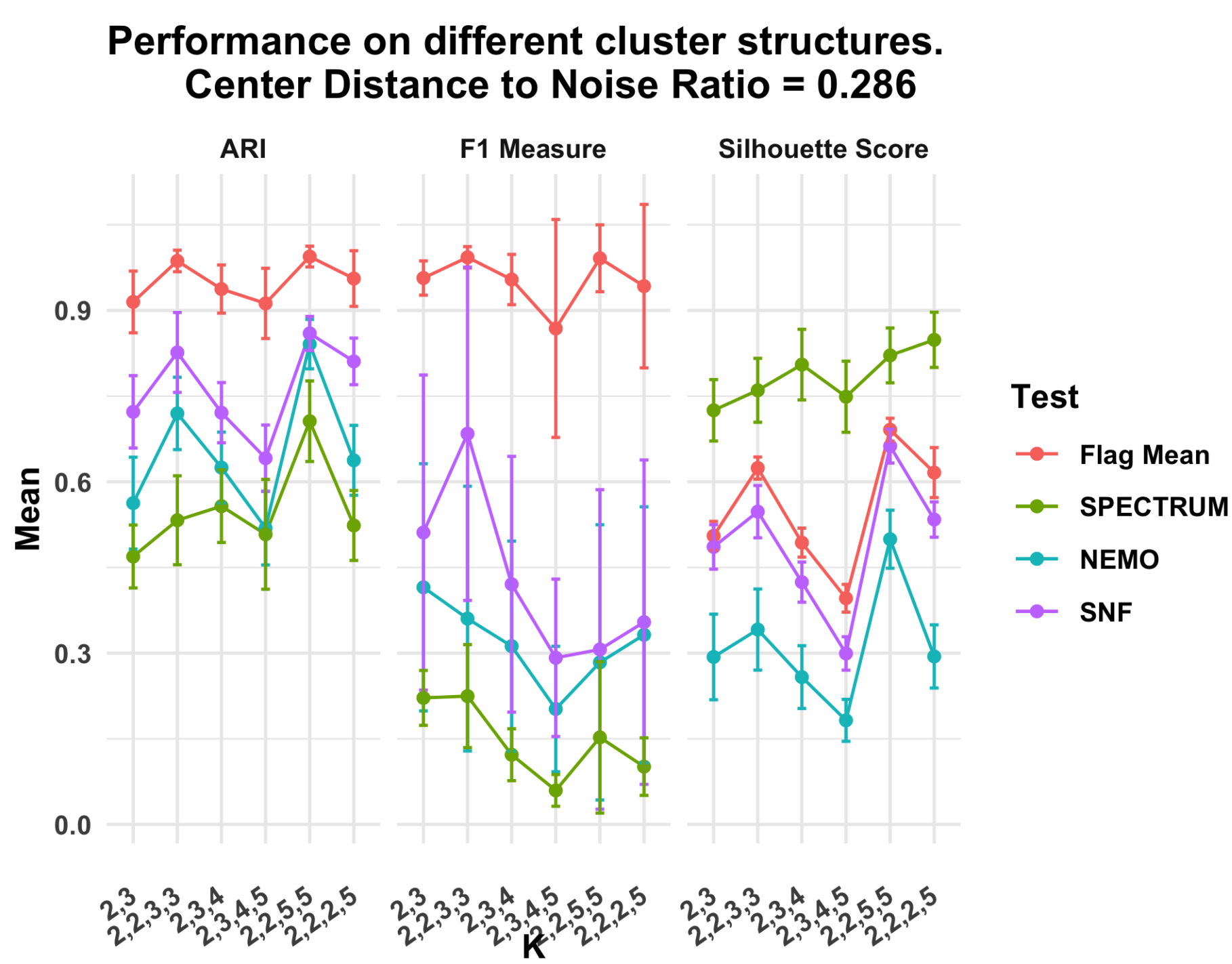

Fig. S2: Results from 5000 simulations with a minimum center distance to noise ratio of 0.286 ($\tau = 7$). Averages are displayed as dots with error bars showing ± one standard deviation.

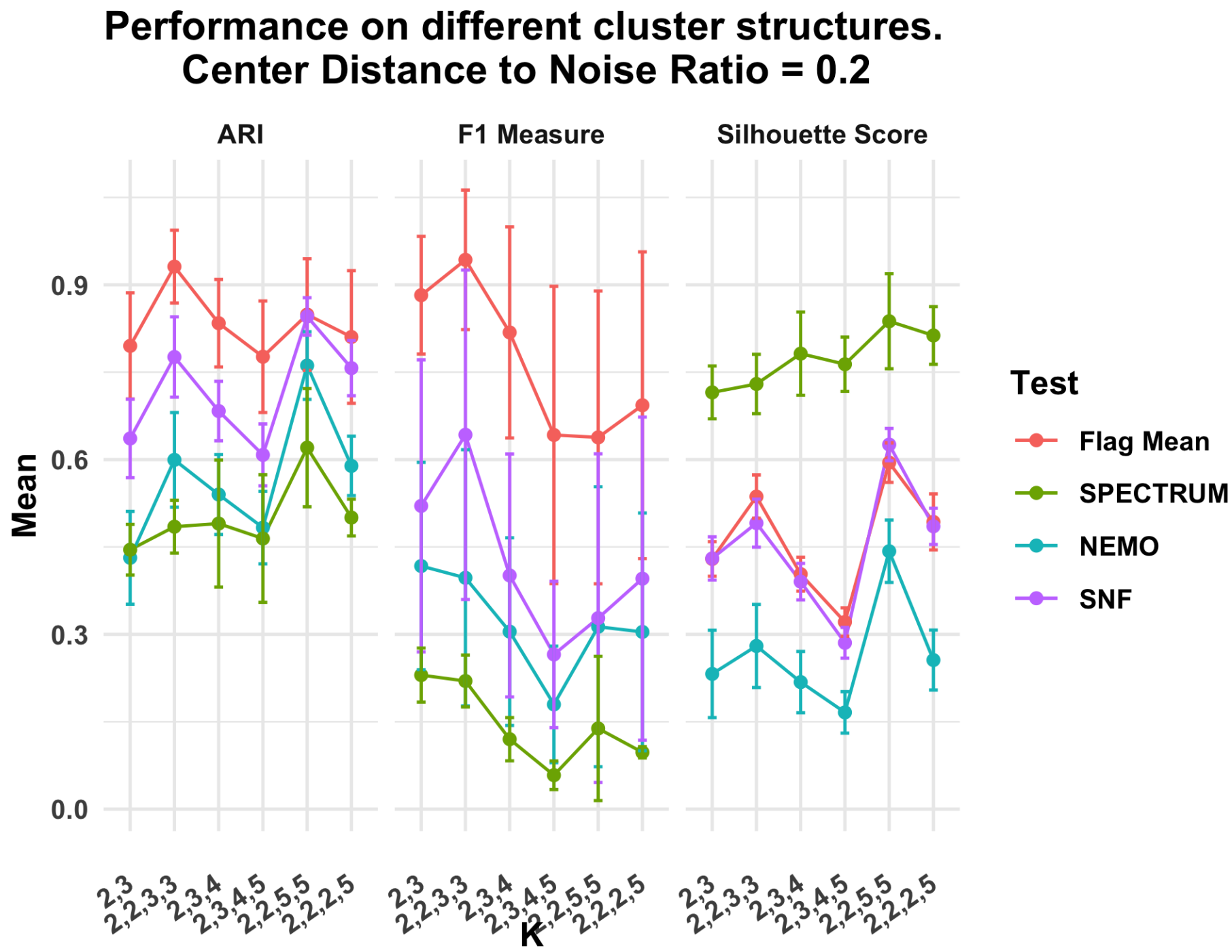

Fig. S3: Results from 5000 simulations with a minimum center distance to noise ratio of 0.2 ($\tau = 10$. Averages are displayed as dots with error bars showing $\pm$ one standard deviation.

Changing number of features across views

The following plots summarize the performance of the proposed methods and the competitors' when the number of features changes across views. In general, we again see mediocre performance in our proposed methods' silhouette scores. Our methods outperform NEMO and SNF in both F1 and ARI in 2-, 3-, and 4-view studies. Spectrum gives slightly higher F1 and ARI means in some settings of 2-view studies, but not in 3- or 4-view studies.

In the 2-view simulations (Figure S4), we see that Spectrum has a slight edge over our methods when the number of features is larger in the view with more clusters, specifically when P=(30,90) or P=(30,120). They lose this when the number of features are closer together (P=(60,90) or P=(90,60)) and they perform terribly when higher features are with lower number of groups (P=(120,30)). In this case it underestimates the number of true clusters to 2 consistently (not shown).

In the 3-view simulations (Figure S5) we see our proposed methods definitively outperform the competitors in ARI and F1 measure. NEMO and SNF have fairly consistent average performances with high variability in the F1 measures. Spectrum does not have much variability around it's averages, but the F1 measures vary widely between simulation settings. Oddly, Spectrum has its best F1 measures when the lowest number of features are with the view with the most clusters, which is the opposite of what we saw in the simulation above.

We see similar performances in the 4-view setting as we did in the 3-view setting (Figure S6). Our methods outperform the others in ARI and F1 measure, but still has a middling silhouette score. NEMO and SNF have very consistent performances in all settings, but have slightly higher variability in their F1 measures compared to their ARI and silhouette scores. Spectrum has hardly any variability in any measure within a setting but the most variability in averages among the different settings of P. It has the lowest F1-measure consistently but the highest silhouette score. We see that our F1-measures are more variable than the competitors but much higher on average.

Changing noise across views

We see a similar pattern when changing the amount of extra noise as we did when changing the number of features. Spectrum performs very well in the 2 view setting, but not as well in the 3 or 4 view settings. Its silhouette score is always the highest of the methods. NEMO and SNF both have very consistent mean performances no matter the number of views, but both always have highly variable results. Our proposed methods perform consistently well and the different levels of noise do not impact their average scores. The number of views impacts the variability around the average scores, but the average ARI and F1 measure are above 0.90 for all three settings despite the middling silhouette scores.

In the 2-view simulations (Figure S7) we see that Spectrum performs very well, nearly perfectly in fact, when the levels of noise are closer to one another. When the levels of noise are very different from one another, i.e. when RN=(2,7) or RN=(7,2), Spectrum's ARI

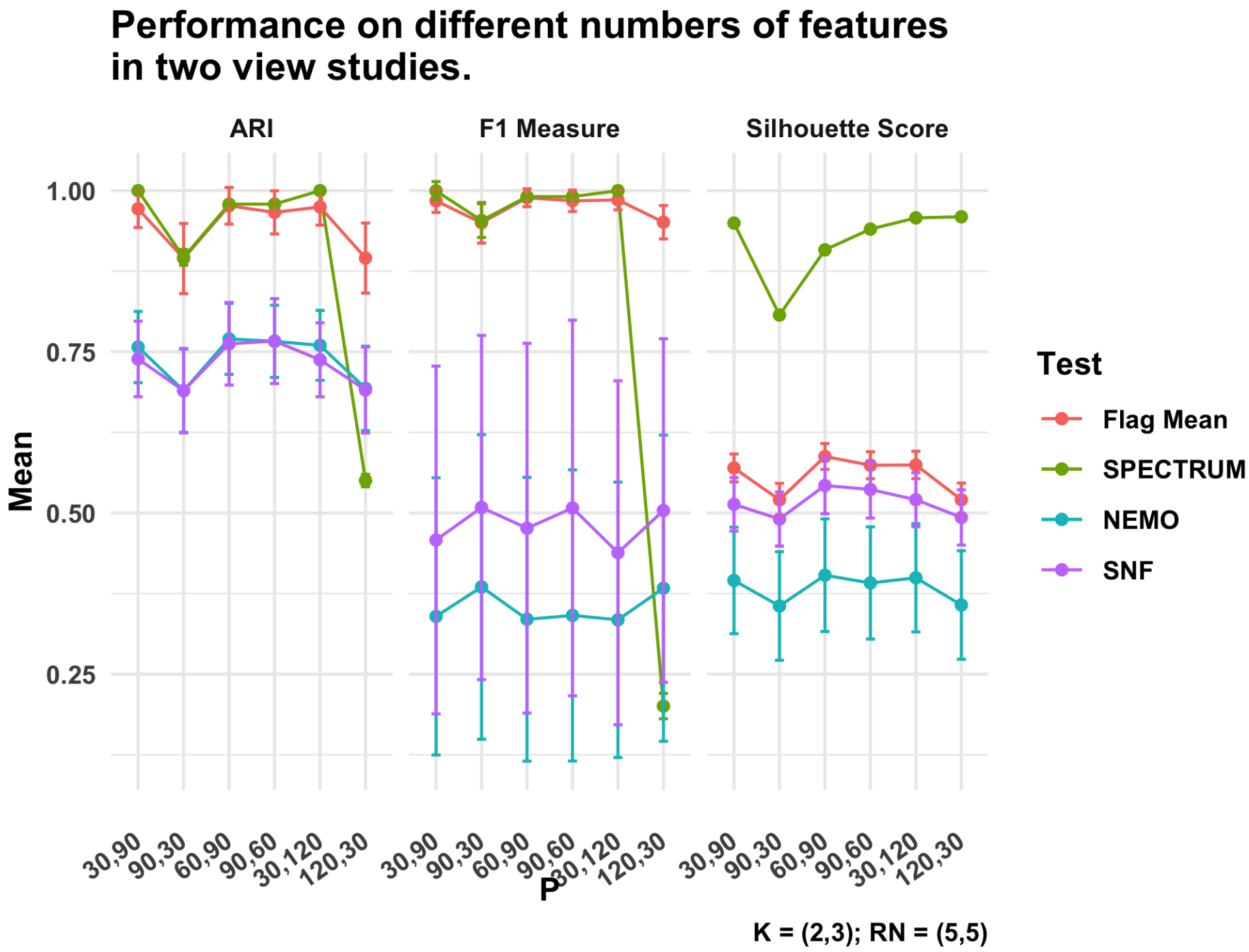

Fig. S4: Results from 5000 simulations on multiple 2-view studies. P is the number of features within each view. K is the number of groups in each view, and RN is the amount of extra noise added.

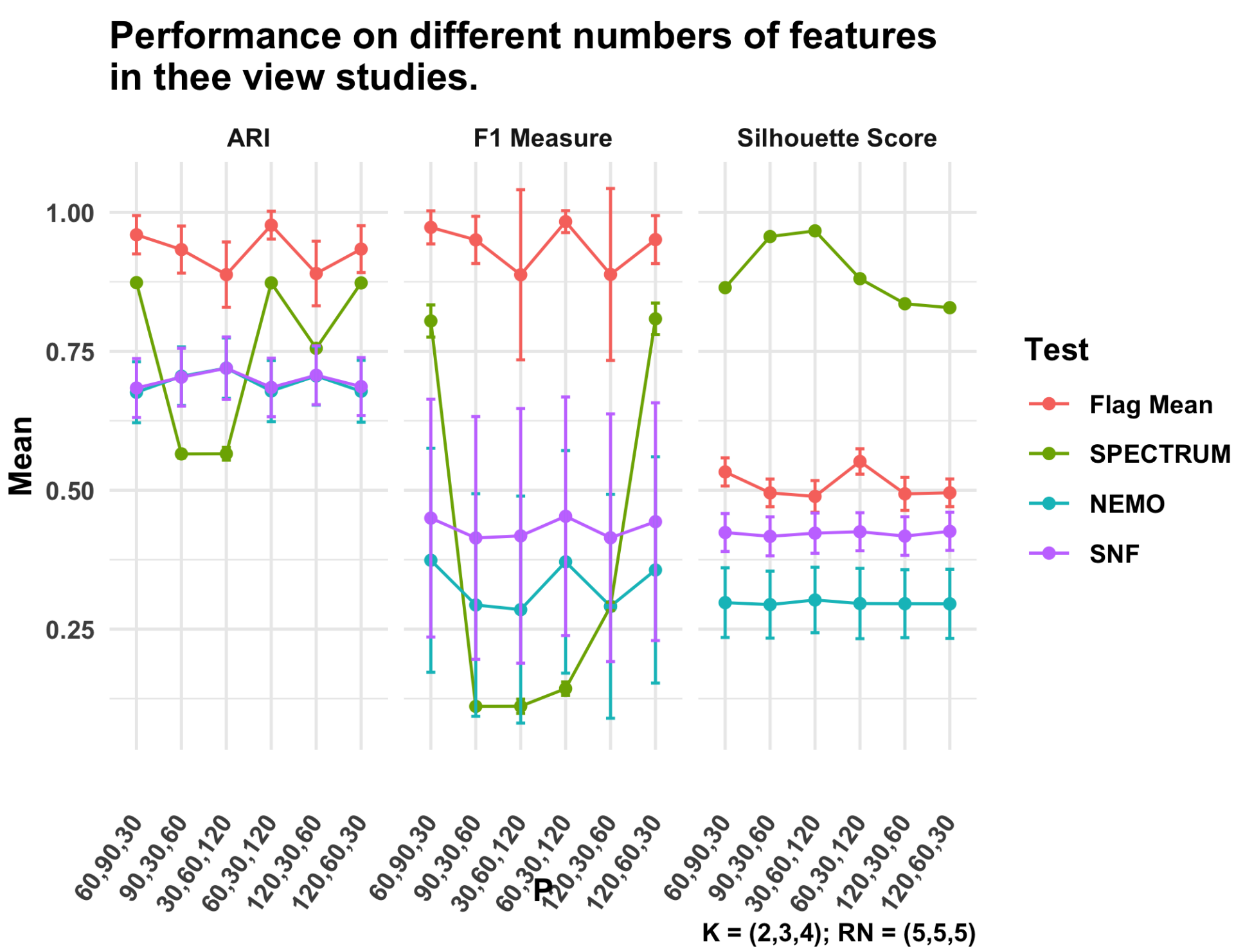

Fig. S5: Results from 5000 simulations on multiple 3-view studies. P is the number of features within each view. K is the number of groups in each view, and RN is the amount of extra noise added.

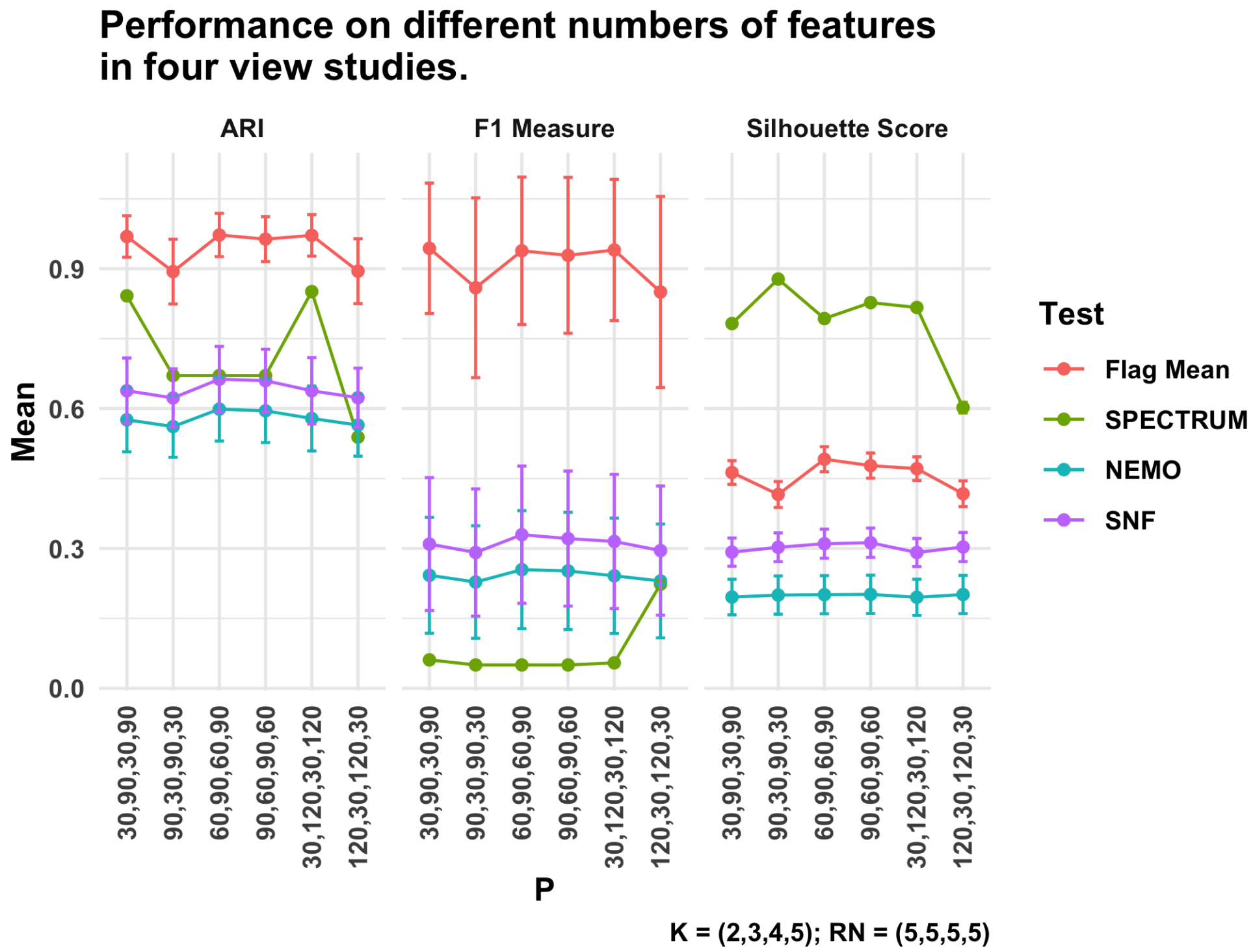

Fig. S6: Results from 5000 simulations on multiple 4-view studies. P is the number of features within each view. K is the number of groups in each view, and RN is the amount of extra noise added.

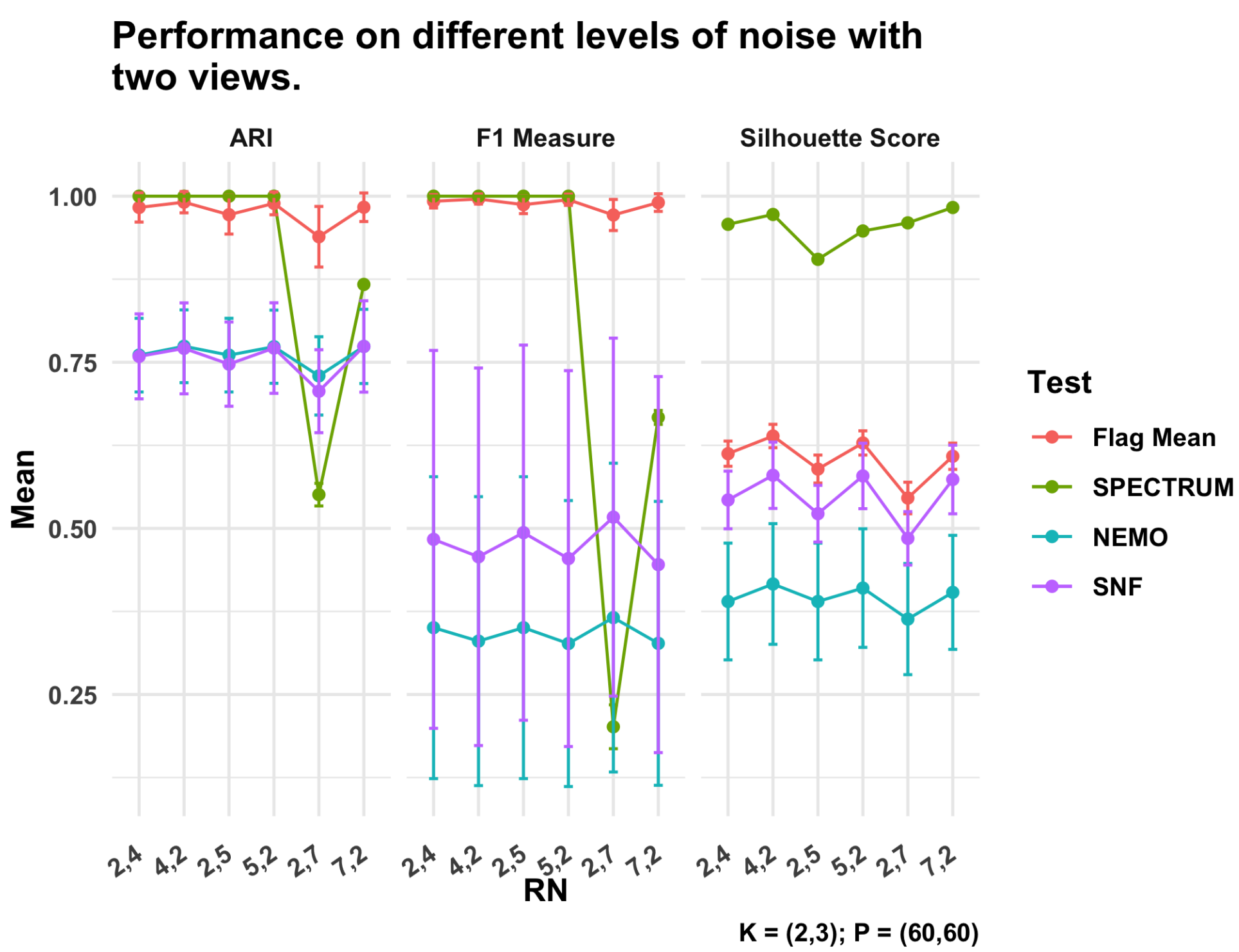

Fig. S7: Results from 5000 simulations on multiple 3-view studies. RN is the amount of extra noise added to each view. K is the number of groups in each view, and P is the number of features within each view.

and F1 measures drop significantly. Despite the low ARI and F1 measures in these settings, Spectrum has its highest silhouette scores. Again NEMO and SNF have fairly consistent average performances for all measures in all settings. The F1 measures of the two methods are extremely variable though. Our proposed methods give very consistent average ARI and F1 measures. Our silhouette measures are middling and seem to dip a little when there is more noise in the view with more clusters.

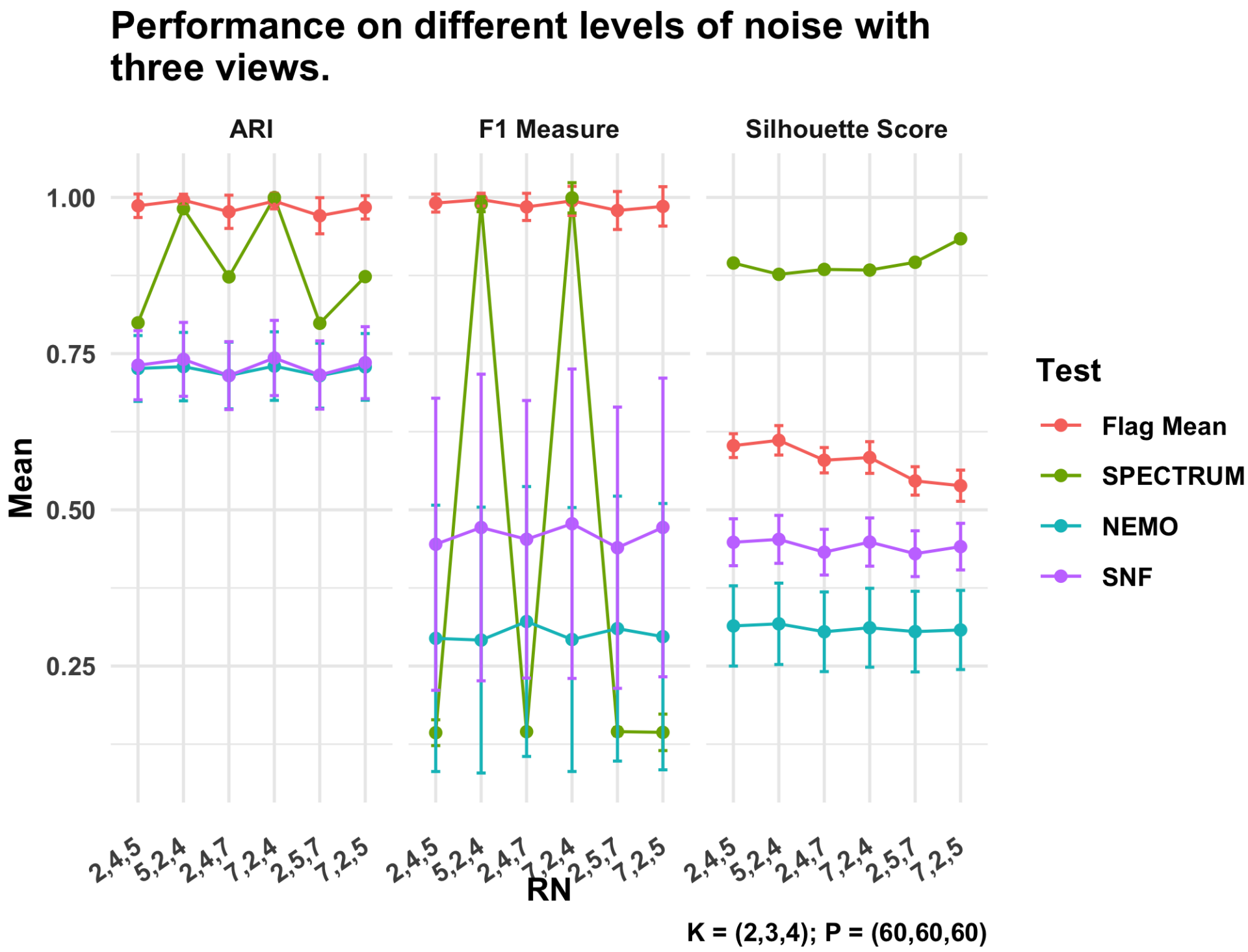

Fig. S8: Results from 5000 simulations on multiple 3-view studies. RN is the amount of extra noise added to each view. K is the number of groups in each view, and P is the number of features within each view.

In the 3-view simulations we see similar performances from our methods as we did in the 2-view setting (Figure S8). The averages are very consistent and high for the predictive measures (ARI and F1) with average silhouette scores around 0.60. There is low variability around these averages as well, especially compared to NEMO and SNF. NEMO and SNF both have lower average silhouette scores and ARI than our proposed methods with relatively low variability around this average. Their average F1 measures are consistent across simulation settings but they are highly variable within each setting. Spectrum performs very interestingly. When RN=2 ($\tau = 2$) in the view with K=3, Spectrum gives a very high ARI and F1 measure until there is higher total noise in the system (RN=(7,2,5), far right on the x-axis of Figure S8). The silhouette scores are unaffected by these changes, and the highest silhouette score comes from this study with the most noise and worst F1 measure. We aren't exactly sure why Spectrum behaves this way, but it is reassuring to see that our methods are consistent.

In the 4-view setting (Figure S9) we see that the our methods beat the competitors in every setting under every measure. All of the methods have a relatively consistent average silhouette and F1 measure. Our F1 measures are more variable than they were in the previous two simulation studies, but this is somewhat expected to come with more views. Spectrum, again, gives the best silhouette score in all settings. The F1 measures of Spectrum are terribly low with almost no variability between or within settings. This indicates that Spectrum can not handle 4-views with different group sizes, regardless of the noise levels. The ARI from Spectrum is reasonably high again when the lowest noise is associated with the view with the largest number of clusters, but it never reaches the performance of our methods. Both NEMO and SNF give very consistent performances with low variability. The variability within setting is the lowest it has been for both methods, but the average performances in all three measures is also the lowest.

Selecting K in Joint Clustering Space

The Figures S10, S11, S12, and S13 show the findings in the 2-, 3-, and 4-view simulation studies we ran.

We see many indices perform well in our 2-view simulation (Figure S10). There are 4 distinct groups in this simulation setting as indicated by the solid red line. The ladle estimators both do well in estimating k in the joint space from L^R, and Ladle PCA does well with both joint spaces. The *marriot*, *ptbiserial*, *sdbw*, *tau*, and *traceW* all do very well with both joint spaces. The *traceCovW* index does better with the L^R joint space, but underestimates in the flag mean's joint space. The other indices either heavily overestimate k (e.g. *ccc*, *cindex*) or underestimate k (e.g. *beal*, *gap*).

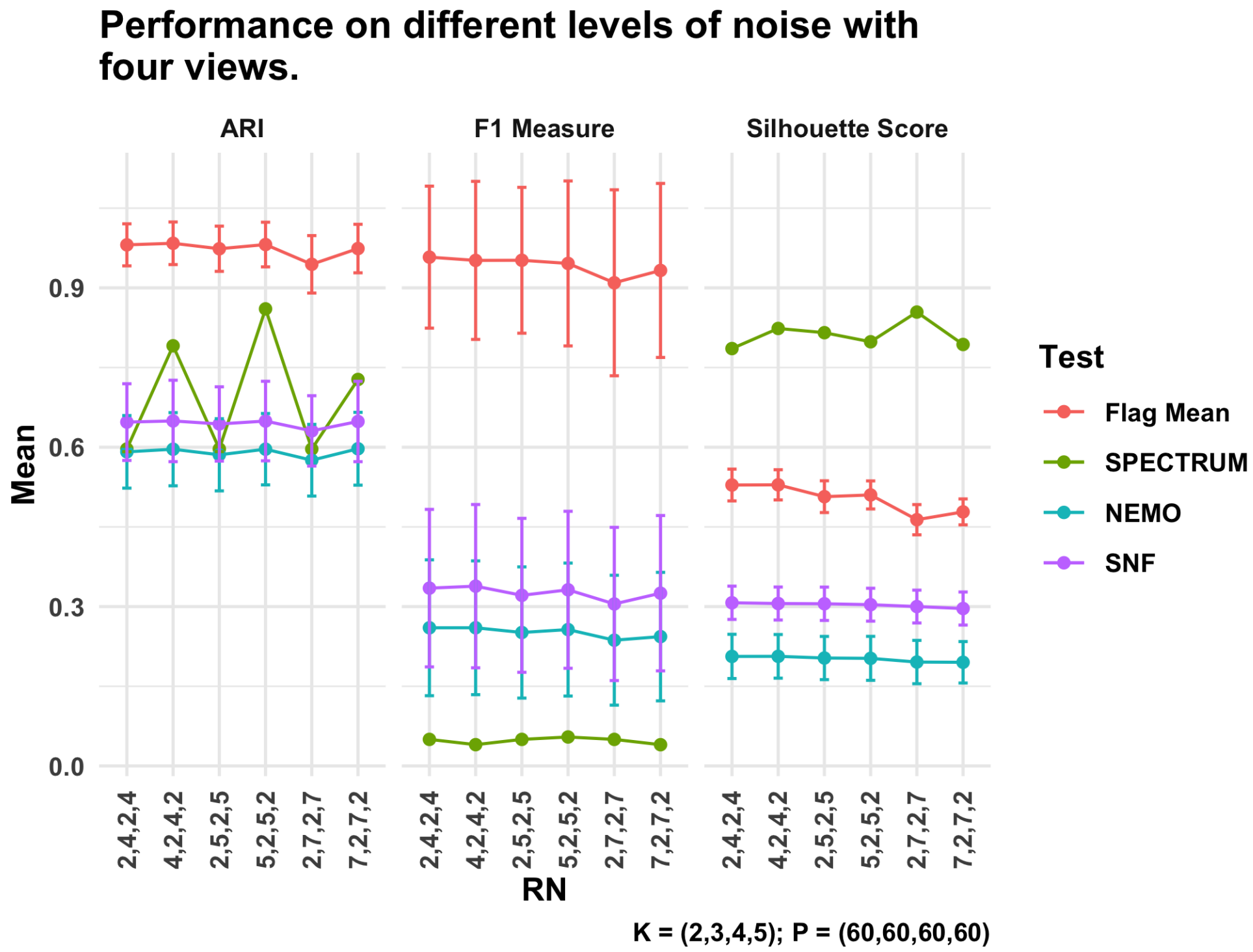

Fig. S9: Results from 5000 simulations on multiple 4-view studies. RN is the amount of extra noise added to each view. K is the number of groups in each view, and P is the number of features within each view.

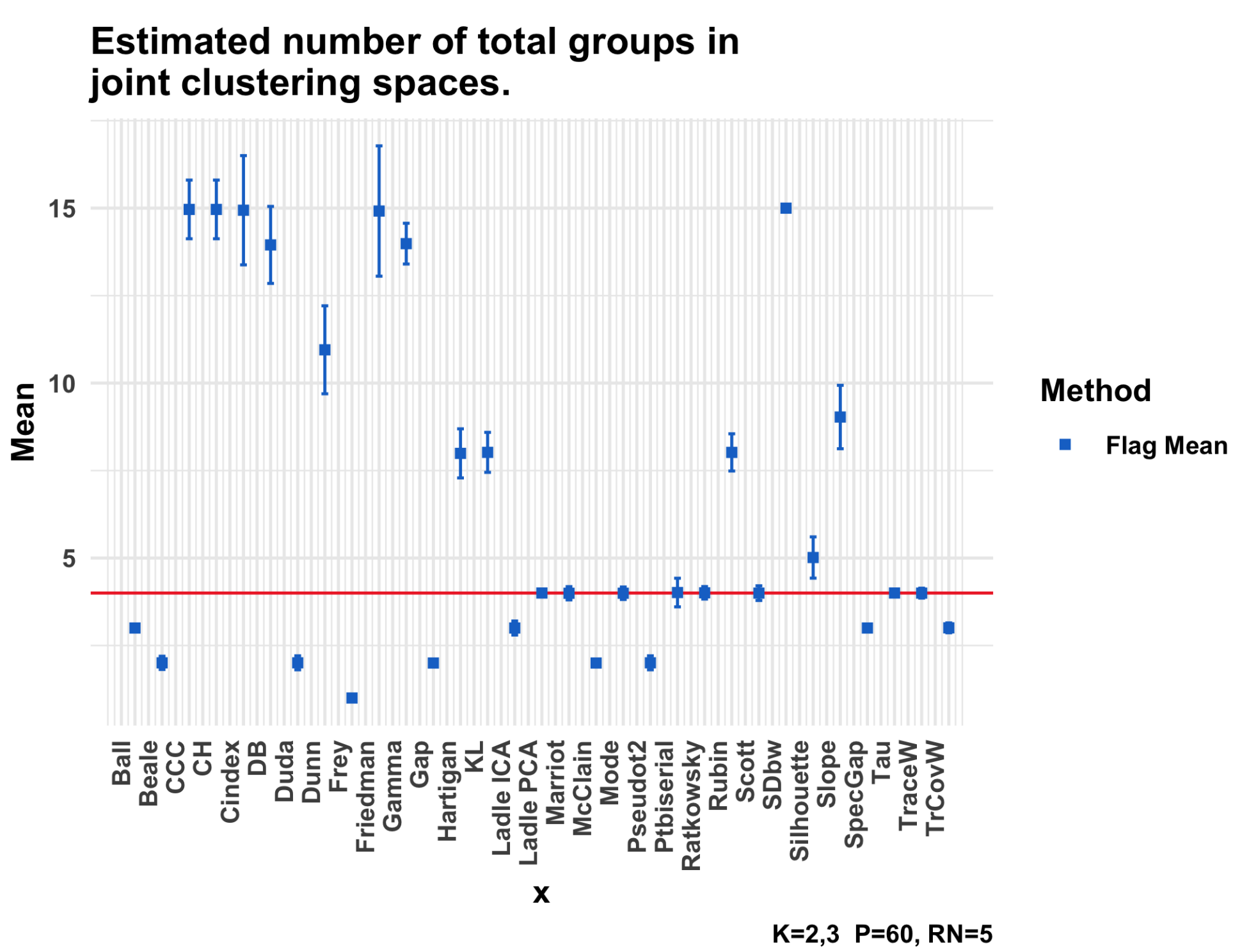

Fig. S10: Results from 1000 simulations on 2-view studies. K is the number of groups in each view. P is the number of features within each view. RN is the amount of extra noise added to each view. The solid red line indicates the true number of clusters in the joint space.

In the simulation with 3-views (Figures S11 and S12) we see that many of the indices that estimated low in the 2-view study give lower estimates again, some do not even change their estimate (e.g. *ball*, *beal*, *gap*). The *hartigan*, *kl*, *rubin*, *silhouette*, *tau*, and *traceW* indices all correctly estimate $k = 10$. Other estimates here are much closer than they were in the 2-view study, but we wish to note that indices such as *ccc* and *ch* always estimate many groups, but never accurately.

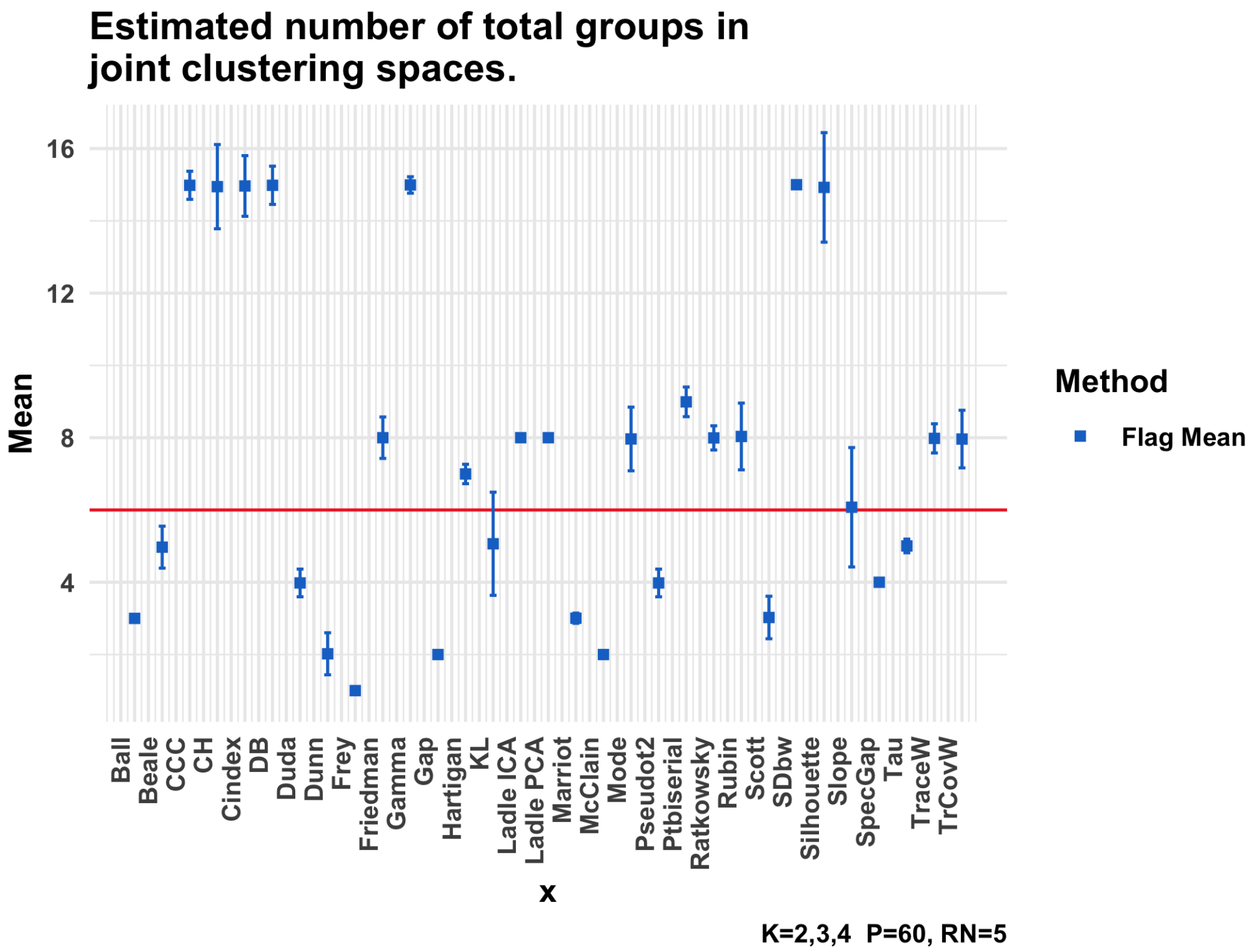

Fig. S11: Results from 1000 simulations on 3-view studies. K is the number of groups in each view. P is the number of features within each view. RN is the amount of extra noise added to each view. The solid red line indicates the true number of clusters in the joint space.

In the simulation with 4-views we see the overall worst performance from our tested indices (Figure S13). The *traceW* index is relatively close to the true number of groups along with *traceCovW*, *tau*, and *hartigan*. We see that *hartigan* is the only index to estimate k correctly.

In summary, there isn't one method that performed best under all scenarios. We recommend focusing on indices that use the within, W, and between, B, sums of squares matrices as their main sources of information. The *traceW* index performs well under all scenarios, but some such as the *hartigan* and *kl* do slightly better when more groups exist.

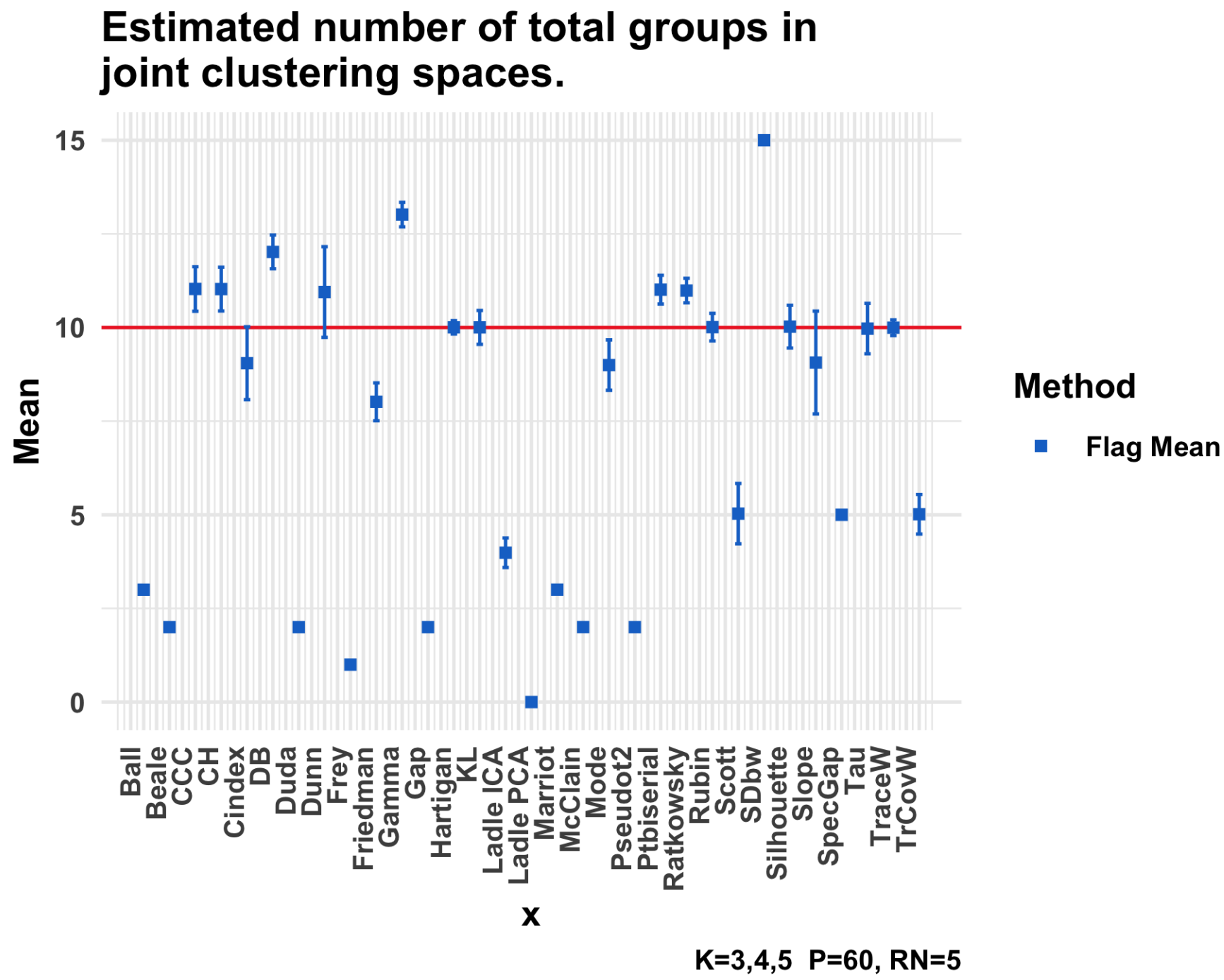

Fig. S12: Results from 1000 simulations on 3-view studies. K is the number of groups in each view. P is the number of features within each view. RN is the amount of extra noise added to each view. The solid red line indicates the true number of clusters in the joint space.

	**Transcriptome Groups**		
**Microbiome Groups**	**1**	**2**	**3**
**1**	14	13	67
**2**	8	9	105

Table S1. The 2 clusters estimated in the microbiome data by the 3 clusters estimated in the transcriptome data

	**Transcriptome Groups**		
**Combined Groups**	**1**	**2**	**3**
**1**	0	22	0
**2**	22	0	0
**3**	0	0	67
**4**	0	0	105

Table S2. The 4 clusters in the joint clustering spaces (Combined Groups) by the 3 clusters from the transcriptome data. Note that the labels in the Combined Groups comes from the L^R clustering, but is equivalent to the groups from the flag mean.

VAP Cluster Summaries

The two microbiome groups split the three transcriptome groups roughly in half (Table S1). There are arguably 6 groups within this set. We believe that the smaller two transcriptome groups are not split in our final joint clustering for two reasons. First, the sample size within transcriptome groups 1 and 2 are fairly small, so it is difficult to separate them based on the noisy clustering information from the microbiome. Second, as we see in both joint clustering spaces (Figures S14), the differences within groups 1 and 2 are not that large, meaning that the transcriptome groups are more similar than the microbiome groups are different.

There were many differences among our estimated groups in the transtricptome and microbiome (Table S3). The p-value from the ANOVA F-statistic was less than 0.05 in 35,197 of the transcripts out of 36,816 (95.6%) after an FDR correction. The were many differences between the individual groups after the Tukey's corrections. We see a very different result from the microbiome. Only 144 of the 1056 microbes had p-value from the ANOVA F-statistic less than 0.05 (13.6%), and there were very few differences among the clusters. We see the most differences between clusters 3 and 4, which is the opposite of what we saw in the transcriptome, suggesting the microbiome differences are driving the separation of these clusters and the transcriptome is driving the separation of the others.

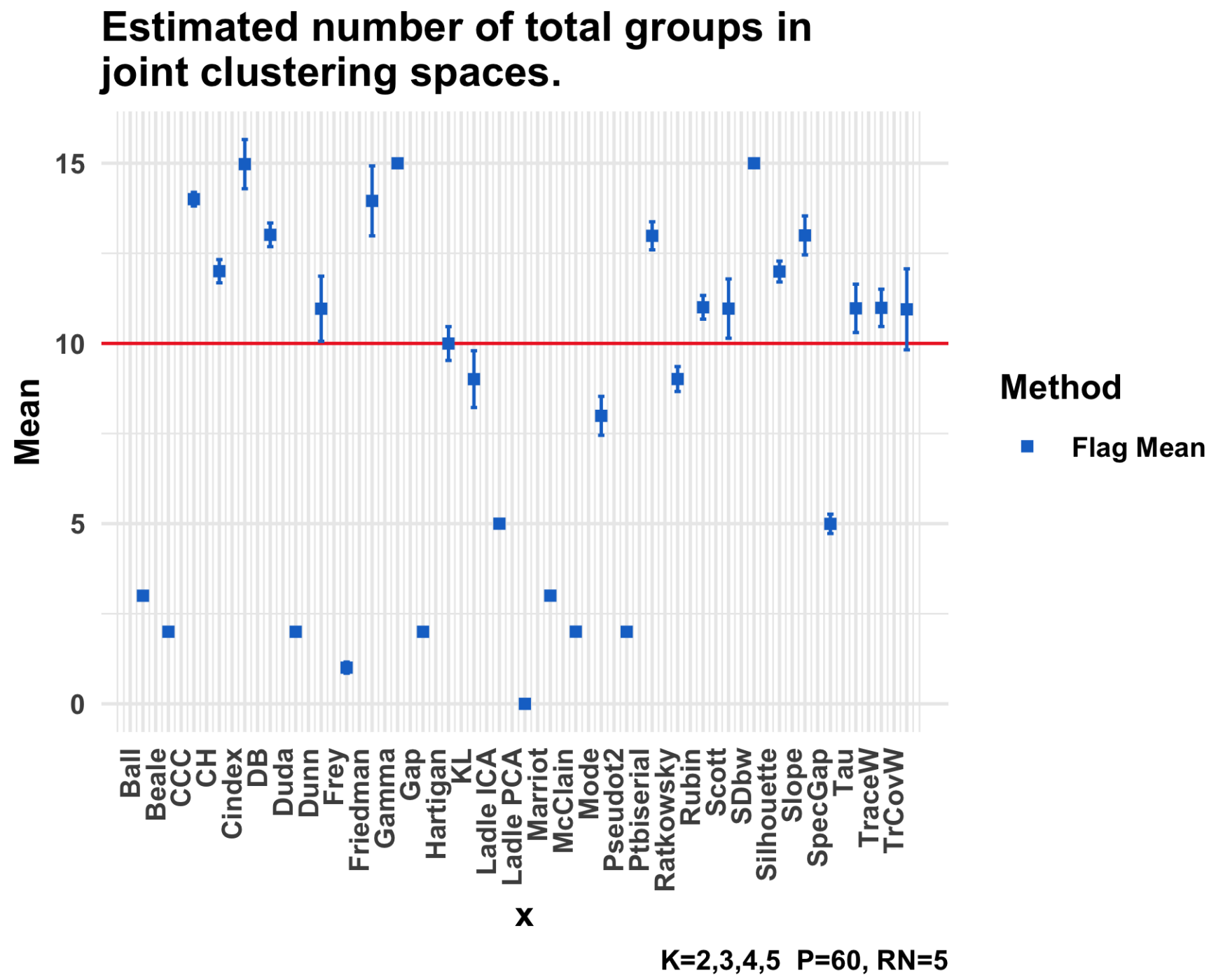

Fig. S13: Results from 1000 simulations on two view studies. K is the number of groups in each view. P is the number of features within each view. RN is the amount of extra noise added to each view. The solid red line indicates the true number of clusters in the joint space.

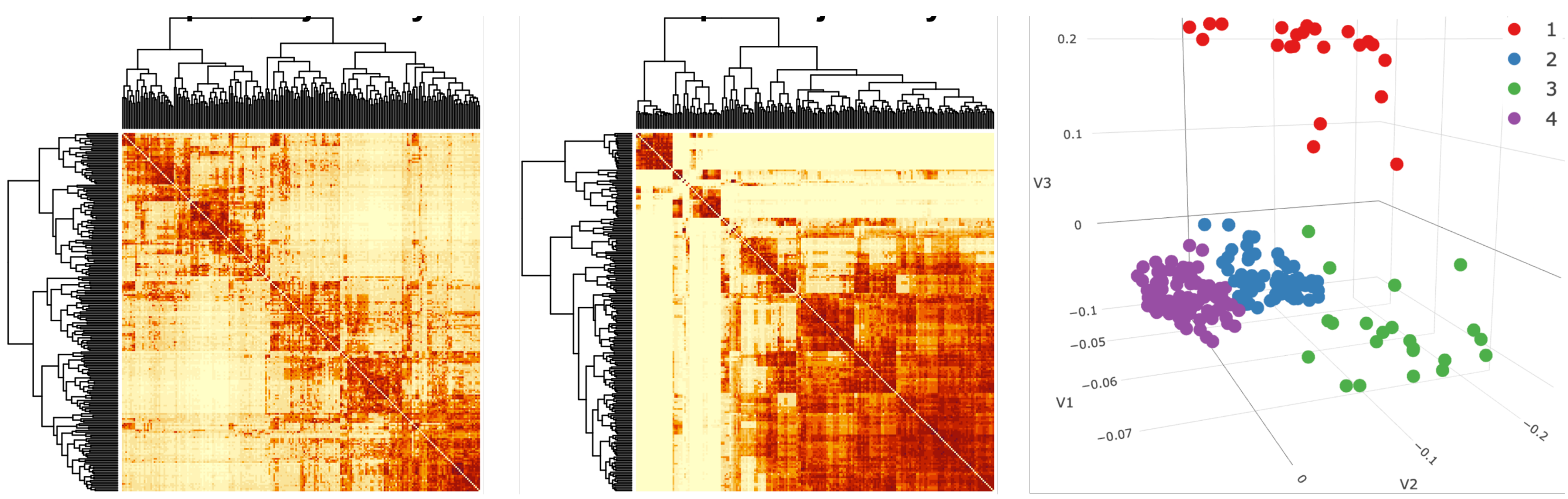

Fig. S14: Heatmaps of the adjacency matrices from the a) microbiome data and b) transcriptome data. The final estimated clusters are displayed within the first three left singular vectors of the flag mean in c).

TCGA breast cancer analyses

The following supplementary analyses support the revised TCGA breast cancer results. They summarize the view-level contributions to the flag mean coordinates, the molecular characterization of clusters 3 and 4, and the robustness checks for the high-risk PFI signal.

METABRIC Validation Cohort

To evaluate whether the clustering patterns observed in TCGA generalize to an independent breast cancer cohort, we also applied the competing methods and our Flag Mean procedure to the METABRIC data. METABRIC is a large breast cancer resource assembled from over 2,000 clinically annotated primary fresh-frozen tumors collected in the UK and Canada, with matched copy number and gene expression profiling and long-term clinical follow-up. In the original study, the cohort was analyzed as discovery and validation sets of 997 and 995 primary tumors, respectively, and integrated CNA–expression analysis revealed molecular subgroups with distinct clinical outcomes. A later extension of the same resource added targeted mutation profiling for 2,433 primary tumors and further emphasized that

Estimated Clusters	Percent of Transcriptome	Percent of Microbiome
**Flag Mean**		
2-1	86.6	0.5
3-1	80.7	0.8
3-2	93.2	2.7
4-1	80.4	7.1
4-2	93.2	3.4
4-3	2.3	13.4
**Spectrum**		
2-1	93.2	25.0
**NEMO**		
2-1	10.0	60.2
3-1	5.5	27.3
4-1	11.5	36.9
5-1	16.8	5.7
6-1	33.9	35.2
7-1	12.1	48.9
8-1	25.0	41.5
9-1	21.3	11.4
3-2	4.1	13.6
4-2	12.7	15.9
5-2	18.8	2.3
6-2	39.9	11.9
7-2	10.2	59.1
8-2	27.7	30.1
9-2	19.6	13.1
4-3	38.3	9.1
5-3	15.0	1.1
6-3	31.5	13.1
7-3	5.6	22.2
8-3	19.8	12.5
9-3	15.5	6.8
5-4	2.5	0
6-4	44.8	5.1
7-4	11.1	38.2
8-4	33.4	17.0
9-4	27.0	7.4
6-5	6.3	0.6
7-5	18.1	2.3
8-5	8.6	0.6
9-5	17.7	1.1
7-6	34.5	42.0
8-6	25.6	13.1
9-6	39.7	6.3
8-7	24.5	30.1
9-7	23.0	19.3
8-9	26.4	8.5
**SNF**		
2-1	38.3	67.6
3-1	9.3	36.4
3-2	24.4	35.8

Table S3. Summary of the ANOVA Tukey's HSD tests in the VAP study. The 'Percent of' columns shows the percent of the 36,816 transcripts or 176 microbes with p-values ¡ 0.05 from a Tukey's HSD test between estimated clusters within each view.

METABRIC captures clinically relevant inter- and intra-tumor heterogeneity with substantial survival annotation (Curtis et al., 2012; Pereira et al., 2016). Because TCGA and METABRIC are independent breast cancer cohorts with related but non-identical molecular

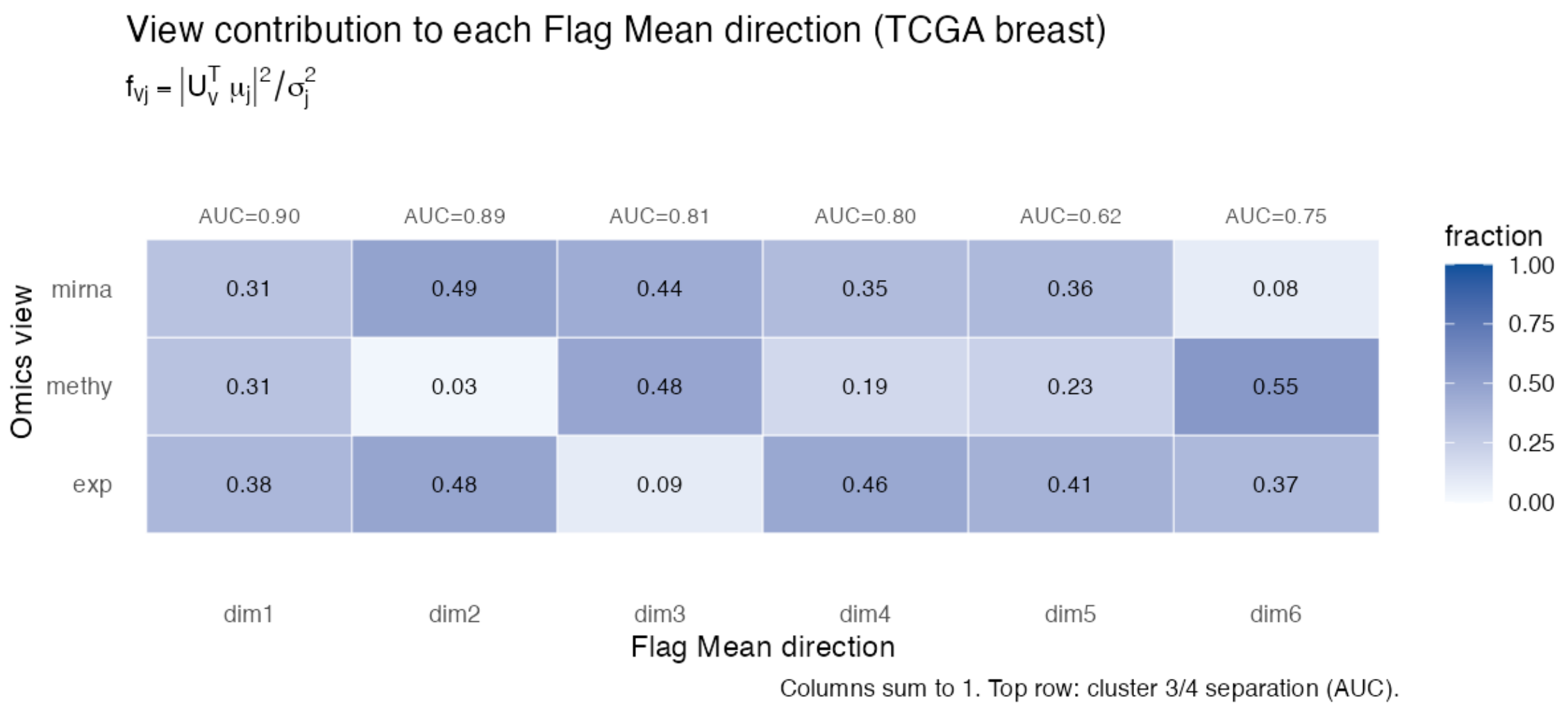

Fig. S15: View fractions for each TCGA flag mean direction. For flag direction μ_j, the fraction from view v is $f_{vj} = c_{vj}/\sigma_j^2$, where $c_{vj} = \|U_v^\top \mu_j\|_2^2$. AUC values annotate separation of clusters 3 and 4 from all other patients. The leading direction has AUC 0.90 and comparable mass across expression, methylation, and miRNA.

layer	feature	effect	t	P.Value	FDR	direction
expression	PRDX1	-0.554	-4.7	3.67e-6	0.0306	down in 3/4
expression	OVCH2	0.917	4.68	4.09e-6	0.0306	up in 3/4
expression	C1orf111	1.037	4.63	5.15e-6	0.0306	up in 3/4
expression	PPAPDC1A	-1.424	-4.23	2.99e-5	0.076	down in 3/4
expression	PDCL3	-0.343	-4.23	3e-5	0.076	down in 3/4
expression	CYB5A	-0.763	-4.19	3.42e-5	0.076	down in 3/4
expression	TSPAN15	-0.66	-4.19	3.55e-5	0.076	down in 3/4
expression	HTRA3	-1.043	-4.15	4.07e-5	0.076	down in 3/4
expression	HELQ	0.281	4.14	4.37e-5	0.076	up in 3/4
expression	DYNLL1	-0.352	-4.08	5.61e-5	0.076	down in 3/4
expression	TFCP2L1	1.414	4.07	5.63e-5	0.076	up in 3/4
expression	NFX1	0.308	4.07	5.84e-5	0.076	up in 3/4
expression	BGN	-0.748	-4.06	5.94e-5	0.076	down in 3/4
expression	CEP68	0.341	4.06	5.96e-5	0.076	up in 3/4
expression	CLDN14	-0.968	-4.03	6.74e-5	0.0801	down in 3/4
expression	LOC441177	0.767	3.98	8.11e-5	0.0801	up in 3/4
expression	PLEK2	-0.688	-3.98	8.15e-5	0.0801	down in 3/4
expression	TFR2	-1.279	-3.96	8.93e-5	0.0801	down in 3/4
expression	APOC2	-1.031	-3.96	9.05e-5	0.0801	down in 3/4
expression	ZP1	-1.012	-3.94	9.59e-5	0.0801	down in 3/4
expression	NCRNA00152	-0.629	-3.94	9.89e-5	0.0801	down in 3/4
expression	ZNF33B	0.453	3.93	9.94e-5	0.0801	up in 3/4
expression	BANF1	-0.427	-3.92	1.05e-4	0.0801	down in 3/4
expression	AREG	1.718	3.91	1.09e-4	0.0801	up in 3/4
expression	TUBA1C	-0.422	-3.89	1.21e-4	0.0801	down in 3/4

Table S4. Leading adjusted expression features for the contrast of flag mean clusters 3 and 4 against all other TCGA breast cancer clusters. Effects are from the PAM50 plus clinical adjusted model.

and clinical structure, METABRIC provides a useful external validation setting for assessing whether any advantage of the proposed method persists beyond the TCGA analysis.

A particularly important feature of METABRIC is that its original integrative taxonomy was strongly driven by copy-number-associated expression structure. Curtis et al. reported that CNAs dominated the expression landscape and used cis-associated CNA-driven signals to define ten integrative clusters with distinct prognosis, while the follow-up METABRIC study again emphasized that CNAs explaining expression in cis were enriched for driver biology and underlay the 'IntClust' classification (Curtis et al., 2012; Pereira et al., 2016). This makes METABRIC a stringent benchmark for multi-view clustering methods, especially those intended to recover biologically meaningful cancer subtypes and survival-relevant structure from genomic data.

layer	feature	effect	t	P.Value	FDR	direction
methylation	cg12519676	1.718	4.26	2.61e-5	0.131	up in 3/4
methylation	cg12015443	1.35	4	7.72e-5	0.193	up in 3/4
methylation	cg05296099	1.612	3.76	2e-4	0.198	up in 3/4
methylation	cg23591302	1.531	3.67	2.74e-4	0.198	up in 3/4
methylation	cg06413087	1.207	3.66	2.9e-4	0.198	up in 3/4
methylation	cg15912086	1.433	3.65	2.98e-4	0.198	up in 3/4
methylation	cg20929387	1.382	3.64	3.11e-4	0.198	up in 3/4
methylation	cg26975184	1.559	3.6	3.54e-4	0.198	up in 3/4
methylation	cg17095936	0.834	3.52	4.89e-4	0.198	up in 3/4
methylation	cg01791371	1.337	3.5	5.16e-4	0.198	up in 3/4
methylation	cg03262885	1.404	3.44	6.48e-4	0.198	up in 3/4
methylation	cg00662647	1.567	3.44	6.48e-4	0.198	up in 3/4
methylation	cg18261069	-1.232	-3.44	6.56e-4	0.198	down in 3/4
methylation	cg23115390	1.094	3.43	6.7e-4	0.198	up in 3/4
methylation	cg02461341	1.152	3.42	6.99e-4	0.198	up in 3/4
methylation	cg01965874	1.245	3.39	7.69e-4	0.198	up in 3/4
methylation	cg02109189	1.263	3.38	7.94e-4	0.198	up in 3/4
methylation	cg19916740	1.169	3.34	9.22e-4	0.198	up in 3/4
methylation	cg17926950	-1.012	-3.33	9.37e-4	0.198	down in 3/4
methylation	cg26864714	1.469	3.32	9.87e-4	0.198	up in 3/4
methylation	cg21643429	1.054	3.32	9.88e-4	0.198	up in 3/4
methylation	cg21418534	1.19	3.32	0.001	0.198	up in 3/4
methylation	cg18045100	1.255	3.31	0.00102	0.198	up in 3/4
methylation	cg17187762	1.261	3.29	0.00109	0.198	up in 3/4
methylation	cg01992382	1.22	3.29	0.0011	0.198	up in 3/4

Table S5. Leading adjusted methylation features for the contrast of flag mean clusters 3 and 4 against all other TCGA breast cancer clusters. Effects are from the PAM50 plus clinical adjusted model.

layer	feature	effect	t	P.Value	FDR	direction
mirna	hsa-mir-135b	0.929	3.14	0.00183	0.274	up in 3/4
mirna	hsa-mir-153-1	0.801	3.13	0.00188	0.274	up in 3/4
mirna	hsa-mir-940	-0.674	-3.09	0.00217	0.274	down in 3/4
mirna	hsa-mir-3677	-0.683	-2.97	0.00318	0.301	down in 3/4
mirna	hsa-mir-1468	0.538	2.89	0.00402	0.304	up in 3/4
mirna	hsa-mir-2115	-0.506	-2.78	0.00573	0.342	down in 3/4
mirna	hsa-mir-1254	-0.318	-2.73	0.00668	0.342	down in 3/4
mirna	hsa-mir-101-2	0.425	2.68	0.00774	0.342	up in 3/4
mirna	hsa-mir-383	0.428	2.63	0.00889	0.342	up in 3/4
mirna	hsa-mir-132	0.339	2.62	0.00906	0.342	up in 3/4
mirna	hsa-mir-155	-0.528	-2.38	0.0179	0.487	down in 3/4
mirna	hsa-mir-92b	-0.451	-2.38	0.0179	0.487	down in 3/4
mirna	hsa-mir-193b	-0.514	-2.38	0.018	0.487	down in 3/4
mirna	hsa-mir-3170	-0.389	-2.29	0.0225	0.487	down in 3/4
mirna	hsa-mir-501	-0.381	-2.29	0.0227	0.487	down in 3/4
mirna	hsa-mir-221	0.434	2.28	0.0229	0.487	up in 3/4
mirna	hsa-mir-3648	-0.463	-2.26	0.0245	0.487	down in 3/4
mirna	hsa-mir-101-1	0.3	2.25	0.0248	0.487	up in 3/4
mirna	hsa-mir-1307	-0.375	-2.25	0.0253	0.487	down in 3/4
mirna	hsa-mir-142	-0.624	-2.24	0.0258	0.487	down in 3/4
mirna	hsa-mir-210	-0.822	-2.21	0.0276	0.497	down in 3/4
mirna	hsa-mir-345	-0.498	-2.13	0.0342	0.588	down in 3/4
mirna	hsa-mir-192	-0.399	-2.09	0.0374	0.597	down in 3/4
mirna	hsa-mir-323b	-0.506	-2.07	0.0388	0.597	down in 3/4
mirna	hsa-mir-425	-0.475	-2.04	0.0419	0.597	down in 3/4

Table S6. Leading adjusted miRNA features for the contrast of flag mean clusters 3 and 4 against all other TCGA breast cancer clusters. Effects are from the PAM50 plus clinical adjusted model.

cluster	n	pfi_events	median_pfi_months	group
1	9	1	NA	other
2	99	15	NA	other
3	59	13	120.6	high-risk (3/4)
4	10	2	NA	high-risk (3/4)
5	10	0	NA	other
6	428	48	138.2	other

Table S7. Cluster sizes, PFI event counts, and median PFI for the final TCGA flag mean clustering.

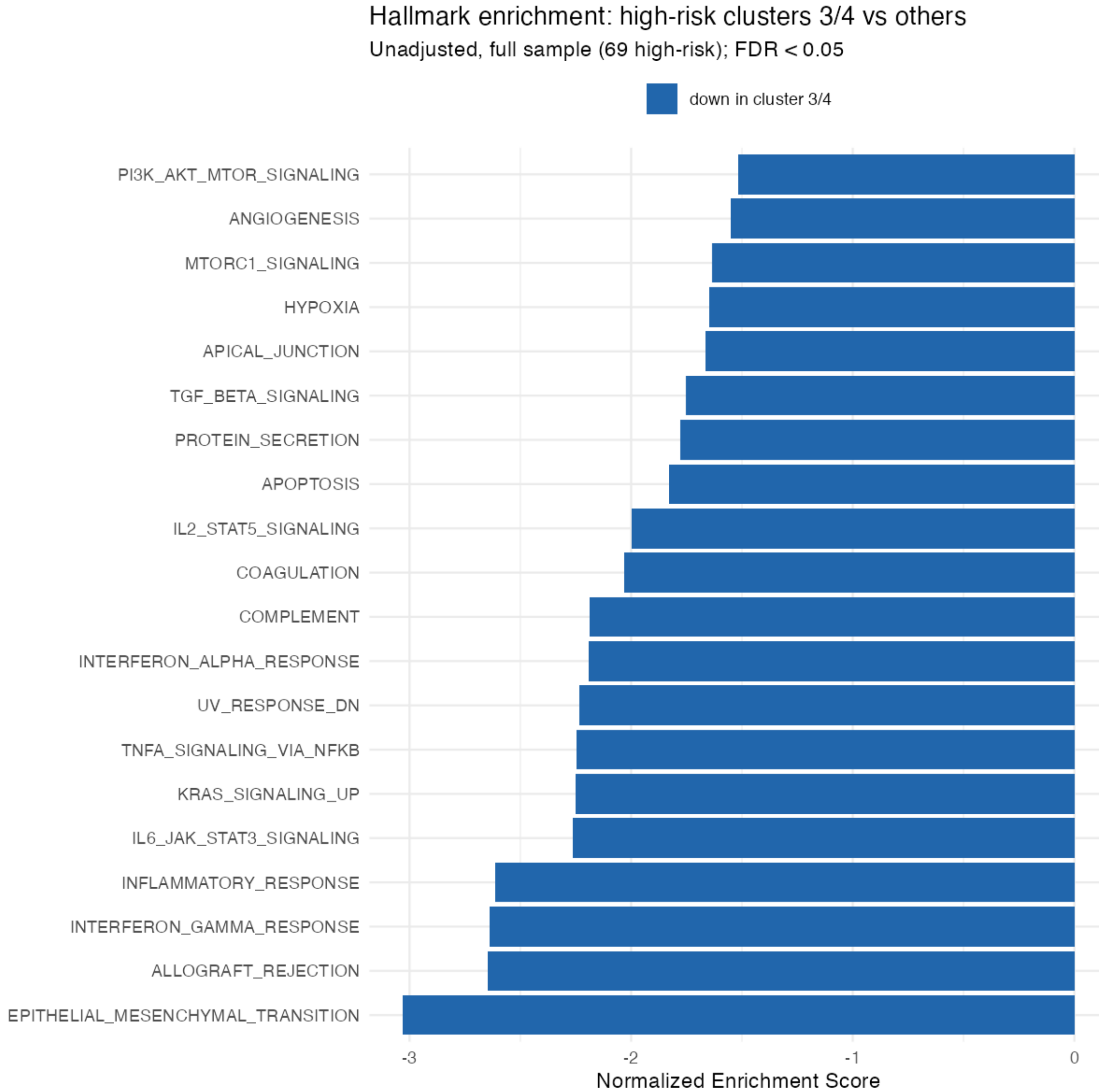

Fig. S16: Hallmark enrichment for the expression contrast of flag mean clusters 3 and 4 against all other clusters in the full-sample unadjusted TCGA analysis. Negative NES values indicate pathways lower in clusters 3 and 4. This analysis provides a molecular annotation of the high-risk clusters and is interpreted alongside the PAM50-adjusted survival models in the main text.

model	term	n	events	hr	ci_low	ci_high	p
cluster 3 alone vs others (n=376, 49 ev) + PAM50	hiC3	376	49	2.80	1.19	6.57	0.018
cluster 4 alone vs others (n=357, 44 ev) + PAM50	hiC4	357	44	5.38	1.26	22.91	0.023

Table S8. PAM50-adjusted Cox models testing each high-risk flag mean cluster alone against all other clusters for TCGA PFI.

model	term	n	events	hr	ci_low	ci_high	p
high-risk, WITHOUT node	highriskCluster34	382	51	3.08	1.43	6.63	0.004
high-risk, WITH node	highriskCluster34	382	51	2.69	1.24	5.87	0.013

Table S9. High-risk Cox model attenuation check with and without nodal status in the same TCGA patients.

Results on METABRIC

The METABRIC results were more mixed than those from TCGA (Table S10). Flag mean estimated 8 clusters, matching SCM in granularity, whereas Spectrum and SNF each collapsed the cohort to 2 clusters and NEMO estimated 5 clusters. In terms of agreement

Method	Performance Metrics			
	**ARI**	**OS log-rank p**	**RFS log-rank p**	**k**
Flag Mean	0.0245	0.0170	0.2301	8
Spectrum	0.0529	0.0575	0.1830	2
SNF	0.0493	0.1074	0.6957	2
NEMO	0.2027	8.46×10^{-9}	5.25×10^{-12}	5
SCM	0.1407	3.34×10^{-7}	3.37×10^{-7}	8

Table S10. Comparison of clustering methods on the METABRIC breast cancer cohort. We report agreement with subtype labels using adjusted Rand index (ARI), log-rank p-values for overall survival (OS) and relapse-free survival (RFS), and the estimated number of clusters (k).

with the reference subtype labels, flag mean had low concordance (ARI =0.0245), lower than Spectrum (0.0529), SNF (0.0493), SCM (0.1407), and especially NEMO (0.2027). Survival separation showed a similar pattern. flag mean produced a nominally significant OS log-rank result (p=0.0170), but its relapse-free survival signal was weak (p=0.2301). By contrast, NEMO and SCM showed much stronger prognostic separation, with OS log-rank p-values of 8.46×10^{-9} and 3.34×10^{-7}, and RFS log-rank p-values of 5.25×10^{-12} and 3.37×10^{-7}, respectively. Spectrum and SNF were intermediate to weak in this cohort. Taken together, these results indicate that the superior PFI performance of flag mean in TCGA did not carry over uniformly to METABRIC. In this external validation setting, methods more closely aligned with the dominant subtype and survival structure of METABRIC, particularly NEMO and SCM, achieved substantially stronger performance. The table-based summary is consistent with the original METABRIC literature, where subtype structure is tightly linked to CNA-driven transcriptional programs and long-term outcome.

One possible interpretation is that METABRIC favors methods that recover the strong shared subtype axis induced by copy-number-driven expression programs, whereas the TCGA analysis benefited more from the finer joint structure captured by the Flag Mean representation. We therefore view the METABRIC experiment as an important complement to the TCGA results: it shows that flag mean is not uniformly dominant across all breast cancer cohorts, but that its behavior depends on the geometry of the joint multi-omic signal present in a given study.

References

G. H. Ball and D. J. Hall. ISODATA, A NOVEL METHOD OF DATA ANALYSIS AND PATTERN CLASSIFICATION. 1965.

E. Beale. *Euclidean cluster analysis.* Scientific Control Systems Limited, 1969.

T. Caliński and J. Harabasz. A dendrite method for cluster analysis. *Communications in Statistics*, 3(1):1–27, Jan. 1974. ISSN 0090-3272. doi: 10.1080/03610927408827101. URL https://www.tandfonline.com/doi/abs/10.1080/03610927408827101. Publisher: Taylor & Francis _eprint: https://www.tandfonline.com/doi/pdf/10.1080/03610927408827101.

M. Charrad, N. Ghazzali, V. Boiteau, and A. Niknafs. NbClust Package. An examination of indices for determining the number of clusters, May 2012. URL https://hal.science/hal-01126138.

C. Curtis, S. P. Shah, S.-F. Chin, G. Turashvili, O. M. Rueda, M. J. Dunning, D. Speed, A. G. Lynch, S. Samarajiwa, Y. Yuan, et al. The genomic and transcriptomic architecture of 2,000 breast tumours reveals novel subgroups. *Nature*, 486(7403):346–352, 2012.

J. C. Dunn†. Well-Separated Clusters and Optimal Fuzzy Partitions. *Journal of Cybernetics*, 4(1):95–104, Jan. 1974. ISSN 0022-0280. doi: 10.1080/01969727408546059. URL https://doi.org/10.1080/01969727408546059. Publisher: Taylor & Francis _eprint: https://doi.org/10.1080/01969727408546059.

T. Frey and H. van Groenewoud. A Cluster Analysis of the D2 Matrix of White Spruce Stands in Saskatchewan Based on the Maximum-Minimum Principle. *Journal of Ecology*, 60(3):873–886, 1972. ISSN 0022-0477. doi: 10.2307/2258571. URL https://www.jstor.org/stable/2258571. Publisher: [Wiley, British Ecological Society].

H. P. Friedman and J. Rubin. On Some Invariant Criteria for Grouping Data. *Journal of the American Statistical Association*, 62(320):1159–1178, Dec. 1967. ISSN 0162-1459. doi: 10.1080/01621459.1967.10500923. URL https://www.tandfonline.com/doi/abs/10.1080/01621459.1967.10500923. Publisher: Taylor & Francis _eprint: https://www.tandfonline.com/doi/pdf/10.1080/01621459.1967.10500923.

A. Fujita, D. Y. Takahashi, and A. G. Patriota. A non-parametric method to estimate the number of clusters. *Computational Statistics & Data Analysis*, 73:27–39, May 2014. ISSN 0167-9473. doi: 10.1016/j.csda.2013.11.012. URL https://www.sciencedirect.com/science/article/pii/S0167947313004507.

M. Halkidi, Y. Batistakis, and M. Vazirgiannis. On Clustering Validation Techniques. *Journal of Intelligent Information Systems*, 17, Oct. 2001. doi: 10.1023/A:1012801612483.

J. A. Hartigan. *Clustering Algorithms.* John Wiley & Sons, Inc., USA, 99th edition, 1975. ISBN 978-0-471-35645-5.

L. Hubert and F. B. Baker. Data Analysis by Single-Link and Complete-Link Hierarchical Clustering. *Journal of Educational Statistics*, 1(2):87–111, June 1976. ISSN 0362-9791. doi: 10.3102/10769986001002087. URL https://doi.org/10.3102/10769986001002087. Publisher: American Educational Research Association.

C. R. John, D. Watson, M. R. Barnes, C. Pitzalis, and M. J. Lewis. Spectrum: fast density-aware spectral clustering for single and multi-omic data. *Bioinformatics*, 36(4):1159–1166, Feb. 2020. ISSN 1367-4803. doi: 10.1093/bioinformatics/btz704. URL https://doi.org/10.1093/bioinformatics/btz704.

L. Kaufman and P. Rousseeuw. *Finding Groups in Data: An Introduction To Cluster Analysis.* Jan. 1990. ISBN 978-0-471-87876-6. doi: 10.2307/2532178. Journal Abbreviation: Wiley, New York. ISBN 0-471-87876-6. Publication Title: Wiley, New York. ISBN 0-471-87876-6.

W. J. Krzanowski and Y. T. Lai. A Criterion for Determining the Number of Groups in a Data Set Using Sum-of-Squares Clustering. *Biometrics*, 44(1):23–34, 1988. ISSN 0006-341X. doi: 10.2307/2531893. URL https://www.jstor.org/stable/2531893. Publisher: [Wiley, International Biometric Society].

C. Luo, J. Xu, Y. Ren, J. Ma, and X. Zhu. Simple contrastive multi-view clustering with data-level fusion. In *Proceedings of the Thirty-Third International Joint Conference on Artificial Intelligence*, volume 5, 2024.

F. H. C. Marriott. Practical Problems in a Method of Cluster Analysis. *Biometrics*, 27(3):501–514, 1971. ISSN 0006-341X. doi: 10.2307/2528592. URL https://www.jstor.org/stable/2528592. Publisher: [Wiley, International Biometric Society].

G. W. Milligan and M. C. Cooper. An examination of procedures for determining the number of clusters in a data set. *Psychometrika*, 50:159–179, 1985. ISSN 1860-0980. doi: 10.1007/BF02294245. Place: Germany Publisher: Springer.

B. Pereira, S.-F. Chin, O. M. Rueda, H.-K. M. Vollan, E. Provenzano, H. A. Bardwell, M. Pugh, L. Jones, R. Russell, S.-J. Sammut, et al. The somatic mutation profiles of 2,433 breast cancers refine their genomic and transcriptomic landscapes. *Nature communications*, 7 (1):11479, 2016.

N. Rappoport and R. Shamir. NEMO: cancer subtyping by integration of partial multi-omic data. *Bioinformatics*, 35(18):3348–3356, Sept. 2019. ISSN 1367-4803. doi: 10.1093/bioinformatics/btz058. URL https://doi.org/10.1093/bioinformatics/btz058.

D. Ratkowsky and G. Lance. A criterion for determining the number of groups in a classification. 1978. URL https://publications.csiro.au/rpr/pub?list=BRO&pid=procite:d307f9e5-5079-4897-b7f7-4232d6ceeccf.

A. J. Scott and M. J. Symons. Clustering Methods Based on Likelihood Ratio Criteria. *Biometrics*, 27(2):387–397, 1971. ISSN 0006-341X. doi: 10.2307/2529003. URL https://www.jstor.org/stable/2529003. Publisher: [Wiley, International Biometric Society].

R. Tibshirani, G. Walther, and T. Hastie. Estimating the number of clusters in a data set via the gap statistic. *Journal of the Royal Statistical Society: Series B (Statistical Methodology)*, 63(2):411–423, 2001. ISSN 13697412. doi: 10.1111/1467-9868.00293. URL https://onlinelibrary.wiley.com/doi/10.1111/1467-9868.00293.

B. Wang, A. M. Mezlini, F. Demir, M. Fiume, Z. Tu, M. Brudno, B. Haibe-Kains, and A. Goldenberg. Similarity network fusion for aggregating data types on a genomic scale. *Nature Methods*, 11(3):333–337, Mar. 2014. ISSN 1548-7105. doi: 10.1038/nmeth.2810.
